# MFN2 deficiency affects calcium homeostasis in lung adenocarcinoma cells via downregulation of UCP4

**DOI:** 10.1002/2211-5463.13591

**Published:** 2023-03-14

**Authors:** Jingjing Zhang, Lifang Pan, Qiang Zhang, Yanyan Zhao, Wenwen Wang, Nengming Lin, Shirong Zhang, Qiong Wu

**Affiliations:** ^1^ Department of Translational Medicine Research Center, Key Laboratory of Clinical Cancer Pharmacology and Toxicology Research of Zhejiang Province, Affiliated Hangzhou First People's Hospital Zhejiang Chinese Medical University Hangzhou China; ^2^ Department of Oncology, Affiliated Hangzhou First People's Hospital, Cancer Center Zhejiang University School of Medicine Hangzhou China; ^3^ Department of Pharmaceutical and Chemical Engineering Zhengzhou Business Technicians Institute China; ^4^ Department of Integrated Chinese and Western Medicine The Cancer Hospital of the University of Chinese Academy of Sciences (Zhejiang Cancer Hospital) Hangzhou China

**Keywords:** calcium homeostasis, lung adenocarcinoma, MFN2, UCP4

## Abstract

Mitofusin‐2 (MFN2) is a transmembrane GTPase that regulates mitochondrial fusion and thereby modulates mitochondrial function. However, the role of MFN2 in lung adenocarcinoma remains controversial. Here, we investigated the effect of MFN2 regulation on mitochondria in lung adenocarcinoma. We found that MFN2 deficiency resulted in decreased UCP4 expression and mitochondrial dysfunction in A549 and H1975 cells. UCP4 overexpression restored ATP and intracellular calcium concentration, but not mtDNA copy number, mitochondrial membrane potential or reactive oxygen species level. Furthermore, mass spectrometry analysis identified 460 overlapping proteins after independent overexpression of MFN2 and UCP4; these proteins were significantly enriched in the cytoskeleton, energy production, and calponin homology (CH) domains. Moreover, the calcium signaling pathway was confirmed to be enriched in KEGG pathway analysis. We also found by protein–protein interaction network analysis that PINK1 may be a key regulator of MFN2‐ and UCP4‐mediated calcium homeostasis. Furthermore, PINK1 increased MFN2/UCP4‐mediated intracellular Ca^2+^ concentration in A549 and H1975 cells. Finally, we demonstrated that low expression levels of MFN2 and UCP4 in lung adenocarcinoma are associated with poor clinical prognosis. In conclusion, our data suggest not only a potential role of MFN2 and UCP4 in co‐regulating calcium homeostasis in lung adenocarcinoma but also their potential use as therapeutic targets in lung cancer.

## Graphical abstract

Mitofusin‐2 (MFN2) is a transmembrane GTPase that modulates mitochondrial function. Here, we found that MFN2 deficiency resulted in decreased UCP4 expression and mitochondrial dysfunction in lung adenocarcinoma. PINK1 may be a key regulator of MFN2/UCP4‐mediated calcium homeostasis. Moreover, low levels of MFN2/UCP4 correlate with poor prognosis in lung adenocarcinoma. Thus, MFN2 and UCP4 may be prospective therapeutic targets for lung cancer therapy.

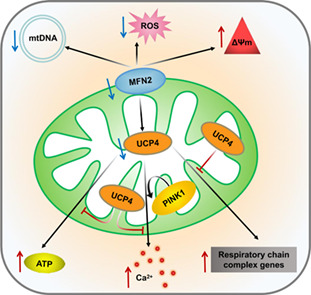

**Abbreviations**
DSS: disease‐specific survival
KD: knockdown
KO: knockout
LUAD: lung adenocarcinoma
MFN2: mitofusin‐2
MMP: mitochondrial membrane potential
mtDNA: mitochondrial DNA
OS: overall survival
PFI: progression‐free interval
PINK1: PTEN‐induced putative kinase protein 1
PPI: protein–protein interaction
qRT–PCR: quantitative real‐time PCR
ROS: reactive oxygen species
TMT: tandem mass tag
UCP4: uncoupling protein‐4

Mitochondria are important organelles that supply cells with ATP, generate reactive oxygen species (ROS), maintain calcium (Ca^2+^) homeostasis, and regulate apoptosis [1]. Altered Ca^2+^ homeostasis contributes to mitochondrial dysfunction in physiology and disease and has been linked to cancer, heart disease, obesity, muscle dysfunction, and pulmonary hypertension [2]. The important role of calcium signaling in lung cancer has also been reported. The expression levels of some specific Ca^2+^ channels and Ca^2+^‐binding proteins are altered in lung cancer and thus affect cell proliferation, metastasis, apoptosis, and tumor growth [3, 4]. The alteration of calcium homeostasis in the endoplasmic reticulum of lung cancer cells is closely related to drug resistance [3]. Therefore, an understanding of the regulation of Ca^2+^ homeostasis is beneficial for further elucidation of the molecular mechanisms underlying lung cancer occurrence and development and will facilitate the development of calcium signaling‐targeted therapies.

Mitofusin‐2 (MFN2), a protein localized in the mitochondrial outer membrane, primarily controls mitochondrial fusion and contributes to mitochondrial homeostasis maintenance [5]. MFN2 dysregulation is associated with various diseases, such as atherosclerosis, Charcot–Marie–Tooth disease, male infertility, obesity, hypertension, diabetes, and cancer [5, 6]. MFN2 plays diverse roles in tumor formation and progression, particularly via its effects on intracellular Ca^2+^ signaling, mitophagy, metabolism, the cell cycle, and apoptosis [5]. Allegra et al. proposed some rational speculations regarding the central role of MFN2 in calcium regulation. On the one hand, as a mitochondria‐ and ER–mitochondria contact‐localized protein, MFN2 may modulate the ER–mitochondria Ca^2+^ flux by interacting with Ca^2+^‐handling proteins. On the other hand, Ca^2+^ acts as an important regulator of cell migration, and the effect of MFN2 on Ca^2+^ flux may also be closely related to tumor metastasis [5, 7]. Moreover, the activity of MFN2 is strictly tissue‐ and tumor‐specific and has completely different effects in different cancers. In lung adenocarcinoma, some studies have implicated MFN2 in exerting tumor‐promoting effects [8, 9], whereas tumor‐suppressing effects of MFN2 have also been reported [10, 11, 12]. Hence, no consistent conclusion regarding the functions of MFN2 in LUAD has been reached.

Uncoupling proteins (UCPs), a family of mitochondrial carrier proteins with the names UCP1–UCP5, are localized in the mitochondrial inner membrane. In particular, UCP4 (also called SLC25A27), which is mainly localized in neurons and neurosensory cells [13, 14], is involved in various aspects of mitochondrial dysfunction, including ATP production, mtDNA copy number regulation, ROS formation, and calcium homeostasis [15, 16, 17]. In addition, UCP4 has a critical function in regulating the proliferation and apoptosis of preadipocytes [18] and chondrocytes [19]. The impact of UCP4 on tumors has not been widely studied. UCP4 expression is correlated with aneuploid tumors and lymph node metastases in breast cancer [20]. UCP4 can also promote ATP synthesis by binding to mitochondrial complex II in neuroblastoma cells [21]. However, whether UCP4 plays a role in LUAD and whether UCP4 and MFN2 coregulate mitochondrial function in lung cancer remain unknown.

In this study, we found that complete MFN2 deficiency resulted in decreased UCP4 expression and mitochondrial dysfunction in A549 and H1975 cells, while UCP4 overexpression restored the intracellular calcium concentration and ATP but not the mtDNA copy number, mitochondrial membrane potential (MMP) or ROS level. Moreover, the protein profiling results demonstrated that MFN2 and UCP4 cooperate in the regulation of multiple different diseases and pathways, including cytoskeletal dynamics, energy metabolism, and calcium homeostasis. Further analyses revealed that MFN2 and UCP4 may regulate calcium homeostasis through the PINK1‐mediated protein–protein interaction (PPI) network. PINK1 could enhance the MFN2/UCP4‐mediated intracellular Ca^2+^ concentration in A549 and H1975 cells. Importantly, we also found that low expression levels of both MFN2 and UCP4 were associated with poor prognosis in LUAD. Thus, these data indicate the possible roles of MFN2 and UCP4 in maintaining PINK1‐mediated calcium homeostasis and suggest prospective therapeutic targets for lung cancer therapy.

## Materials and methods

### Generation of MFN2‐knockout (KO) A549 cells

MFN2‐KO A549 cells were produced by CRISPR/Cas9 genome editing (BRL Medicine Inc., Shanghai, China). Three guide RNA oligos specifically targeting MFN2 (5′‐GCTCTTCTCTCGATGCAACT‐3′, 5′‐CACTTAAGCACTTTGTCACT‐3′ and 5′‐GGAGAGCGCCACCTTCCTTG‐3′, located at MFN2 exon 2) were inserted into pSpCas9(BB)‐2A‐GFP (PX458 was a gift from Feng Zhang; Addgene plasmid #48138) [22] at the BbsI sites and then transfected into A549 cells. FACSAria II (BD Biosciences, San Jose, CA, USA) was used to isolate and culture GFP‐positive single cells in 96‐well plates. Individual MFN2‐KO clones were verified by PCR, DNA sequencing, and western blotting. MFN2‐KO A549 cells were grown in RPMI 1640 medium supplemented with 10% FBS. The primers used to identify the MFN2‐KO mutant were as follows: forward primer, 5′‐CCATTGGGTCTGGCTAA‐3′; reverse primer, 5′‐GCAGTTCCCTTGTCTTCC‐3′ (Table S1).

### Mitochondrial gene arrays and quantitative PCR

Human mitochondria and mitochondrial energy metabolism PCR gene microarray analyses were performed by XYbiotech Company (Shanghai, China). The details of the genes included in the arrays are listed in Table S2. In brief, total RNA was isolated from cells using TRIzol (Invitrogen, Shanghai, China) reagent. The RNA precipitate was washed with 75% ethanol, air‐dried for 5–10 min, and then redissolved in water. DNase I was used to remove genomic DNA from RNA samples, and the samples were then purified with an RNeasy® MinElute™ Cleanup Kit (QIAGEN, Valencia, CA, USA). The quality of RNA was determined by the UV absorption method (A260/A280 ratio of 1.8–2.1) and denaturing agarose gel electrophoresis. cDNA was synthesized using Invitrogen's SuperScript III Reverse Transcriptase, and qRT–PCR was performed with 2X SuperArray PCR Master Mix (Roche, Basel, Switzerland). The expression levels of the target genes were then analyzed with the $2^{-\Delta \Delta C_{t}}$ method.

Quantitative PCR was performed as previously described [23]. Table S1 contains a list of the primers for CDKN2A, NOS2, UCP4, COX6A2, EDN1, TNF, MFN2, and β‐actin that were utilized in this investigation.

### Cell culture, viral vector construction, and infection

Shanghai Institute of Biochemistry and Cell Biology (Shanghai, China) supplied A549 and H1975 cells, which were grown in RPMI 1640 medium supplemented with 10% FBS. The shRNA sequences targeting MFN2 (Table S1) were cloned into the pCLenti‐U6‐shRNA‐CMV‐Neo‐WPRE plasmid. To generate the UCP4‐HA and MFN2‐FLAG expression constructs, UCP4 and MFN2 cDNA were cloned into pLVX‐Puro through digestion with XhoI and XbaI. For lentivirus packaging, lentiviral vectors (pLVX‐Puro, pLVX‐Puro‐UCP4‐HA, pLVX‐Puro‐MFN2‐FLAG, and pLVX‐Puro‐PINK1‐Myc) and envelope/packaging plasmids (pMD2.G and psPAX2) were cotransfected into 293T cells. After 48 h, the viral supernatant was harvested and filtered through a sterile filter (0.45 μm). MFN2‐KO A549 cells and MFN2‐knockdown (KD) H1975 cells were infected with viral supernatant containing polybrene at a concentration of 5 μg·mL^−1^. After 12 h of incubation, puromycin (2 μg·mL^−1^) was used to select stable cell lines.

### Western blotting assays

Western blotting assays were done as reported previously [24]. The primary and secondary antibodies used in the study were as follows: MFN2 (1 : 1000, Cell Signaling Technology, 11925; Danvers, MA, USA), UCP4 (1 : 500, Santa Cruz, sc‐365295; Shanghai, China), NOS2 (1 : 1000, Proteintech, 18985‐1‐AP; Wuhan, China), TNF (1 : 4000, Proteintech, 60291‐1‐Ig), EDN1 (1 : 1000, Proteintech, 12191‐1‐AP), HA (1 : 1000, Cell Signaling Technology, 2367), FLAG (1 : 5000, Sigma, F7425; Shanghai, China), ACTIN (1 : 5000, Proteintech, 23660‐1‐AP), MYC (1 : 5000, Proteintech, 60003‐2‐Ig), PINK1 (1 : 1000, Cell Signaling Technology, 6946), HRP‐conjugated AffiniPure goat anti‐mouse IgG (H + L) (1 : 5000, Proteintech, SA00001‐1), and HRP‐conjugated AffiniPure goat anti‐rabbit IgG (H + L) (1 : 5000, Proteintech, SA00001‐2).

### Mitochondrial copy number determination

Following the manufacturer's instructions, genomic DNA was purified from A549 cells using a PUREGENE DNA Purification kit (QIAGEN). In brief, cell pellets were lysed in Cell Lysis Solution and incubated with RNase A (4 mg·mL^−1^) at 37 °C for 15–60 min. DNA was precipitated with 100% isopropanol and rinsed with 70% ethanol after proteins were precipitated using Protein Precipitation Solution. Finally, the DNA pellets were diluted to 5 ng·μL^−1^ in DNA Hydration Solution after being dissolved. The mitochondrial DNA (mtDNA) copy numbers were determined by qRT–PCR and calculated using the following formulas: ∆*C*_T_ = *C*_T_ (nuDNA)−*C*_T_ (mtDNA) and mtDNA : nuDNA = $2^{\Delta C_{T}}$. The primer sequences used to amplify mtDNA and nuclear DNA (with β‐actin as the internal genomic control) were obtained from the published literature [25] (Table S1).

### Flow cytometry

Flow cytometric analysis was performed using a previously reported method [6]. For intracellular ROS measurement, the cells were treated with 5 μm H_2_DCFDA (D399, Invitrogen) in PBS for 40 min at 37 °C in the dark, collected with 0.05% trypsin–EDTA, suspended in new media, and washed twice with PBS. To detect the intracellular calcium levels, the cells were stained for 30–40 min at 37 °C with the Ca^2+^‐sensitive fluorescent indicator Fluo‐4 AM (2 μm, S1060, Beyotime, Shanghai, China) or Fura‐2 AM (5 μm, S1052, Beyotime). A mitochondrial membrane potential assay kit with JC‐1 (C2006, Beyotime) was used to conduct the JC‐1 assay. Subsequently, flow cytometry analyses were performed using a BD FACS Canto II (BD Biosciences).

### Tandem mass tagging (TMT) proteomics

Tandem mass tagging proteomics was conducted by Jingjie PTM BioLabs (Hangzhou, China). The main steps involved were the processing of protein samples [26], including protein extraction, trypsin digestion, and TMT labeling; HPLC fractionation; LC–MS/MS analysis; and database searching. The detailed experimental procedures have been described previously [27].

### Bioinformatics analysis

#### GO and KEGG pathway annotation

Enrichment analyses were performed at three levels to annotate all identified proteins and screen differentially expressed proteins. These levels included GO classifications (including the biological process, cellular component, and molecular function categories, using the UniProt‐GOA database, http://www.ebi.ac.uk/GOA/), KEGG pathways (using the Kyoto Encyclopedia of Genes and Genomes database, https://www.kegg.jp/), and protein domains (using the InterPro database, https://www.ebi.ac.uk/interpro/).

#### GO/KEGG pathway functional enrichment analyses

For enrichment analyses, which were performed with Fisher's exact test, the *P* values from the bubble plot indicate the functional classifications and pathways of the differentially enriched proteins (*P* < 0.05). The bubble plot displays the results for the top 20 categories with the most significant enrichment.

#### PPI network analysis

Putative PPIs were visualized using the STRING database (version 11.5, https://cn.string‐db.org/). We retrieved all interactions with a confidence score lower than 0.4 (medium confidence).

### Statistical analysis

The data are shown as the means ± SEMs. graphpad prism 8.0 software (San Diego, CA, USA) was used for the data analyses. Student's *t*‐test was utilized to compare the differences across groups. Statistical significance was indicated by *P* value < 0.05.

## Results

### MFN2 is expressed at low levels in lung adenocarcinoma cells

**Fig. 1:**
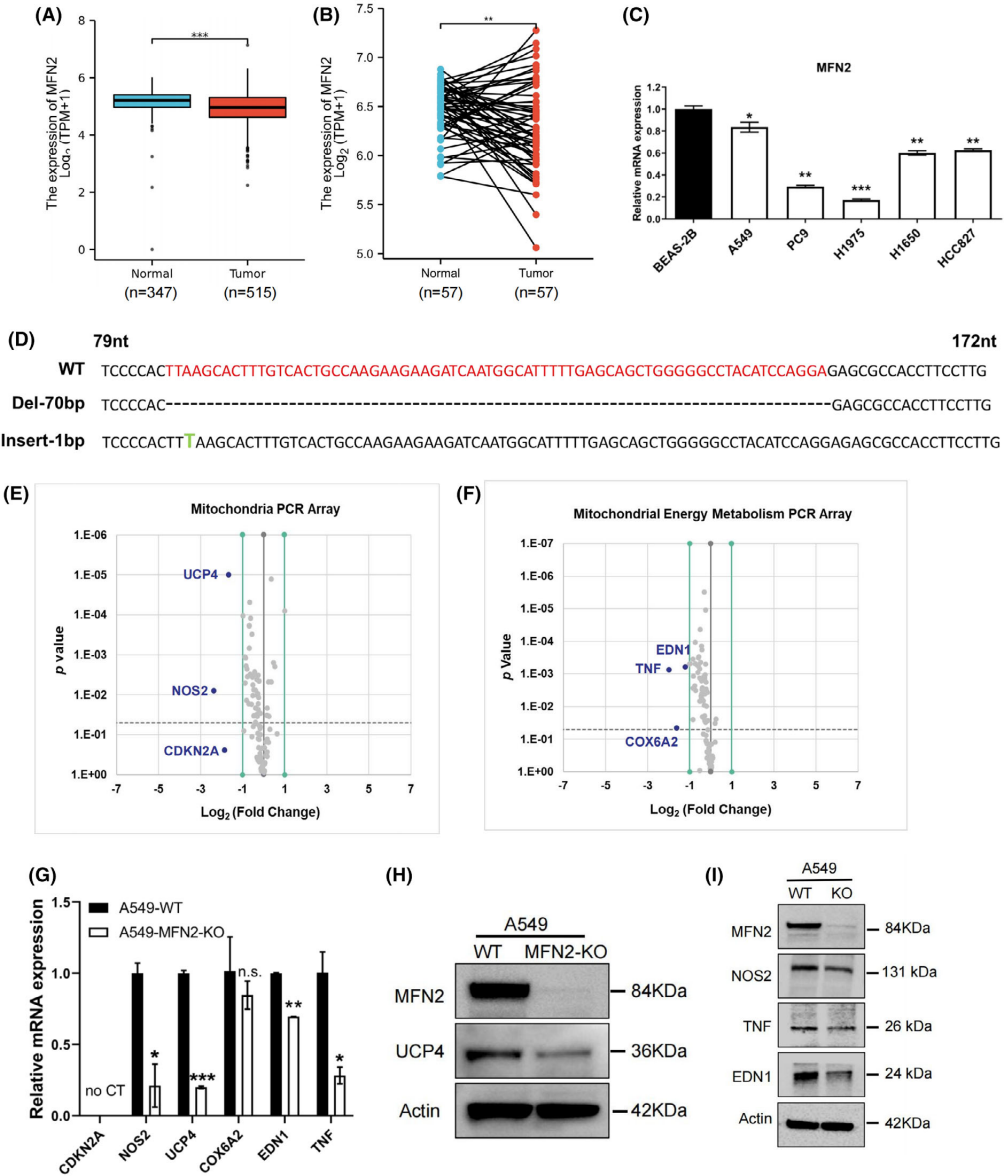
KO of MFN2 reduces the UCP4 mRNA and protein levels in A549 cells. (A, B) Relative MFN2 mRNA expression in unpaired (A, Mann–Whitney *U* test) and paired (B, Student's *t*‐test) samples from the TCGA or GTEx database. (C) The expression of MFN2 in five LUAD cell lines was assessed by qRT–PCR analysis *in vitro*. (D) The sequencing results for mutated alleles of MFN2^−/−^ clone 25 are shown. The KO efficiency was verified using an anti‐MFN2 antibody (H). (E, F) Volcano plot of DEGs identified from three independent samples for the mitochondrial PCR array (E) or mitochondrial energy metabolism PCR array (F). The blue plots represent DEGs, and the gray plots represent non‐DEGs. (G) qRT–PCR validation of DEGs in WT or MFN2‐KO A549 cells. (H) Western blot analysis of MFN2 and UCP4 expression in WT and MFN2‐KO A549 cells. (I) Western blot analysis of NOS2, TNF, and EDN1 expression in WT and MFN2‐KO A549 cells. *DEG*: differentially expressed gene; *P < 0.05, **P < 0.01, ***P < 0.001 (Student's *t*‐test). Values were mean ± SEM of three independent experiments.

The role of MFN2 in lung adenocarcinoma remains highly debated [8, 10, 11]. To determine the expression of MFN2 in LUAD, we first accessed RNA‐seq data in TPM format from ucsc xena (https://xenabrowser.net/datapages/) based on TCGA and GTEx data. The MFN2 expression level in LUAD tissues (*n* = 515) was considerably lower than that in normal lung tissue samples (*n* = 347, *P* < 0.001, Fig. 1A). The mRNA level of MFN2 in 57 paired LUAD tissues was similarly significantly lower than that in adjacent normal tissues (*P* = 0.01, Fig. 1B). Furthermore, we examined the expression of MFN2 in cultured human LUAD cell lines (A549, PC9, H1975, H1650, and HCC827) *in vitro* by real‐time PCR. All five LUAD cell lines had lower MFN2 expression than normal lung epithelial cells (BEAS‐2B; Fig. 1C). These results suggest that MFN2 may be an important tumor suppressor in LUAD. Among the five cell lines, A549 cells exhibited relatively higher MFN2 expression. Therefore, the A549 cell line was selected for subsequent experiments.

### MFN2 deficiency decreases the expression of UCP4 in A549 cells

MFN2 is a mitochondrial dynamics‐related GTPase involved in mitochondrial function and energy metabolism [28]. To investigate the effect of MFN2 on the mitochondrial function of LUAD, CRISPR–Cas9 gene editing was used to generate MFN2‐KO A549 cells. DNA sequencing of clone #25 (62 clones in total) confirmed disruption of the human MFN2 gene, which consisted of a 70‐bp deletion and a 1‐bp insertion in exon 2 that resulted in a frameshift mutation (Fig. 1D). We then validated the MFN2‐KO efficiency by immunoblotting (Fig. 1H). To further identify the molecular differences implicated in mitochondrial function, we analyzed MFN2‐KO A549 cells with a Human Mitochondria PCR Array (90 genes) and a Mitochondrial Energy Metabolism PCR Array (89 genes). Notably, a comparison of the PCR results between MFN2‐WT and MFN2‐KO cells demonstrated that six genes were significantly downregulated (fold change < 0.5): NOS2 (0.2, *P* = 0.00805), CDKN2A (0.28, *P* = 0.24888), UCP4 (0.31, *P* = 0.00001), TNF (0.25, *P* = 0.00076), COX6A2 (0.33, *P* = 0.04589), and EDN1 (0.44, *P* = 0.00063; Fig. 1E,F). The expression changes in these six selected genes were verified by real‐time PCR, which showed that UCP4 exhibited the most markedly reduced expression (Fig. 1G). We also confirmed that the KO of MFN2 resulted in decreased protein expression of UCP4 (Fig. 1H), indicating potential associations between the two genes. Moreover, the protein levels of NOS2, EDN1, and TNF also decreased to different degrees (Fig. 1I), demonstrating the reliability of the PCR array.

### MFN2/UCP4 regulates the intracellular calcium concentration in LUAD cells

**Fig. 2:**
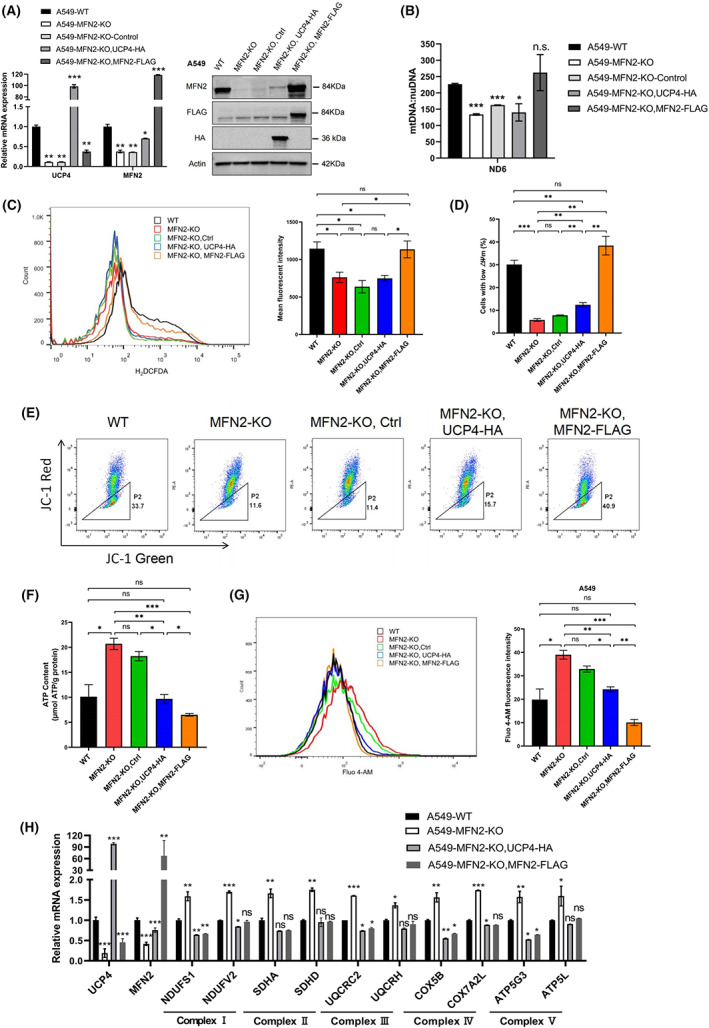
UCP4 overexpression regulates the intracellular calcium concentration in MFN2‐KO A549 cells. (A) qRT–PCR and western blot analysis of UCP4 and MFN2 expression in MFN2‐KO A549 cells stably transfected with UCP4‐HA or MFN2‐FLAG. (B) The mtDNA copy number in MFN2‐KO A549 cells transduced with UCP4‐HA or MFN2‐FLAG was determined by qRT–PCR. (C) The ROS levels in MFN2‐KO A549 cells transduced with UCP4‐HA or MFN2‐FLAG were measured using H_2_DCFDA by flow cytometry. Representative histograms are shown on the left, and a bar graph of the mean fluorescence intensity is displayed on the right. (D, E) The MMP of MFN2‐KO A549 cells transduced with UCP4‐HA or MFN2‐FLAG was determined by JC‐1 staining. (D) shows the proportion of cells with low MMP. (E) indicating representative flow cytometry pictures. (F) The ATP contents in MFN2‐KO A549 cells were measured by an ATP Assay Kit. (G) The Ca^2+^ levels in MFN2‐KO A549 cells overexpressing UCP4‐HA or MFN2‐FLAG were quantified by flow cytometry (histograms on the left). The mean Fluo‐4 AM fluorescence intensity is shown on the right. (H) qRT–PCR analysis of genes related to the mitochondrial respiratory chain complex in MFN2‐KO A549 cells. *P < 0.05; **P < 0.01; ***P < 0.001; n.s., nonsignificant (Student's *t*‐test). Values were mean ± SEM of three independent experiments.

UCP4, a member of the UCP subfamily localized in the inner mitochondrial membrane, is involved in several processes related to mitochondrial dysfunction, including ATP production, mtDNA copy number regulation, ROS formation and calcium homeostasis [15, 16, 17]. Based on the above‐described results, we hypothesized that UCP4 and MFN2 can coregulate mitochondrial function in lung cancer. To test this hypothesis, we first established MFN2‐KO A549 cell lines stably expressing empty vector control, UCP4‐HA or MFN2‐FLAG using a lentiviral system. The overexpression efficiency of UCP4 and MFN2 was verified by RT–PCR and western blotting (Fig. 2A). By qRT–PCR and flow cytometry, we observed not only striking reductions in the mtDNA copy number and ROS level but also increases in the mitochondrial membrane potential (MMP), ATP content, and intracellular calcium concentration in MFN2‐KO A549 cells (Fig. 2B–G). Additionally, we detected a significant increase in the expression of genes related to the mitochondrial respiratory chain complex in MFN2‐KO A549 cells (Fig. 2H), which was consistent with the increased ATP levels. Furthermore, we used siRNA to knock down the expression of MFN2 in different LUAD cell lines (Fig. S1A–D). In addition to HCC827 cells with poor MFN2‐KD efficiency, the results showed that MFN2 KD also resulted in a decreased mtDNA copy number and increased MMP and intracellular calcium concentrations in H1299, H1975, and PC9 cells (Fig. S1E–H). The above data suggest that MFN2 deficiency induces mitochondrial dysfunction in LUAD cells and that this phenomenon is widespread.

**Fig. 3:**
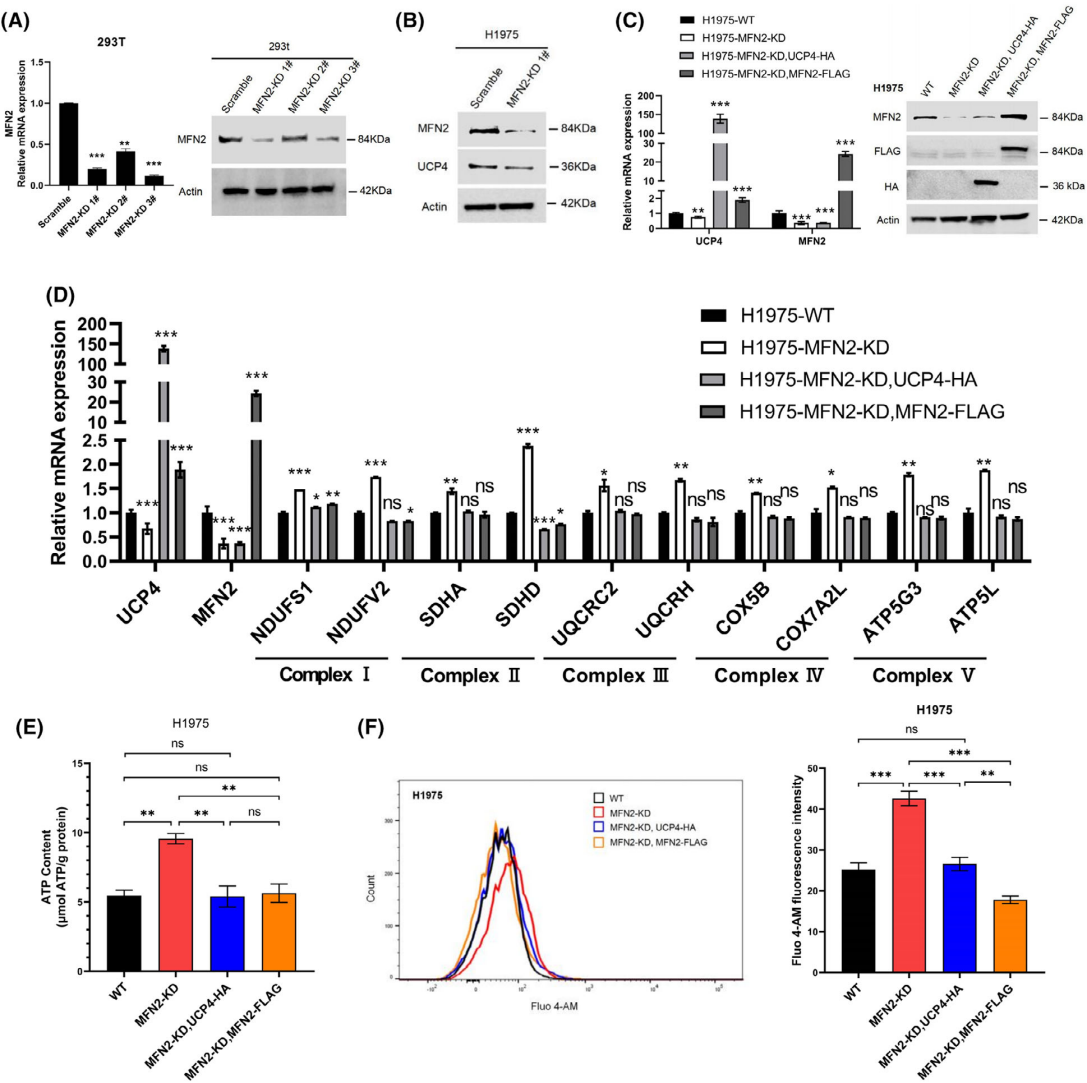
UCP4 overexpression regulates the intracellular calcium concentration in MFN2‐KD H1975 cells. (A) qRT–PCR and western blot analysis of MFN2 expression in 293T cells transduced with MFN2 shRNA#1‐#3. (B) Western blot analysis of MFN2 and UCP4 expression in scramble and MFN2‐KD H1975 cells. (C) qRT–PCR and western blot analysis of UCP4 and MFN2 expression in MFN2‐KD H1975 cells stably transfected with UCP4‐HA or MFN2‐FLAG. (D) qRT–PCR analysis of genes related to the mitochondrial respiratory chain complex in MFN2‐KD H1975 cells. (E) The ATP contents in MFN2‐KD H1975 cells overexpressing UCP4‐HA or MFN2‐FLAG were measured by ATP Assay Kit. (F) The Ca^2+^ levels in MFN2‐KD H1975 cells overexpressing UCP4‐HA or MFN2‐FLAG were quantified by flow cytometry (histograms on the left). The mean Fluo‐4 AM fluorescence intensity is shown on the right. *P < 0.05; **P < 0.01; ***P < 0.001; n.s., nonsignificant (Student's *t*‐test). Values were mean ± SEM of three independent experiments.

Therefore, we subsequently examined whether the overexpression of MFN2 or UCP4 can restore the MFN2‐KO phenotype. As expected, MFN2 overexpression alone completely rescued the effect of mitochondrial dysfunction on the mtDNA level, ROS production, MMP level, ATP generation, expression of mitochondrial respiratory chain‐related genes and intracellular Ca^2+^ concentration (Fig. 2B–H). We also found that UCP4 overexpression caused a large reduction in the ATP level, the expression of mitochondrial respiratory chain‐related genes, and the intracellular calcium concentration, which was measured with the fluorescent Ca^2+^ probes Fluo‐4 AM (Fig. 2G) and Fura‐2 AM (Fig. S1I,J). However, UCP4 overexpression did not affect the mtDNA copy number, ROS level or MMP in MFN2‐KO A549 cells (Fig. 2B–E). To further validate the abovementioned results, we constructed H1975 cells with stable MFN2 KD using shRNA lentivirus (Fig. 3A,B). We found that MFN2 KD caused decreases in UCP4 at the protein and mRNA levels (Fig. 3B,C), which was consistent with the results found in A549 cells. We then established UCP4‐ and MFN2‐overexpressing cell lines based on MFN2‐KD H1975 cells (Fig. 3C). Similar to A549 cells, the overexpression of MFN2 and UCP4 fully restored the elevated ATP levels, mitochondrial respiratory chain‐related gene expression levels, and intracellular Ca^2+^ concentration caused by MFN2 deficiency (Fig. 3D–F). Studies have shown that the primary role of elevated Ca^2+^ is to stimulate oxidative phosphorylation, resulting in higher respiratory chain activity and ATP output [29], as confirmed by our results. Taken together, our data demonstrate that MFN2 and UCP4 cooperate in the regulation of ATP and intracellular calcium homeostasis in lung cancer.

### Proteomic analysis of the MFN2/UCP4 overexpression groups of MFN2‐KO A549 cells

**Fig. 4:**
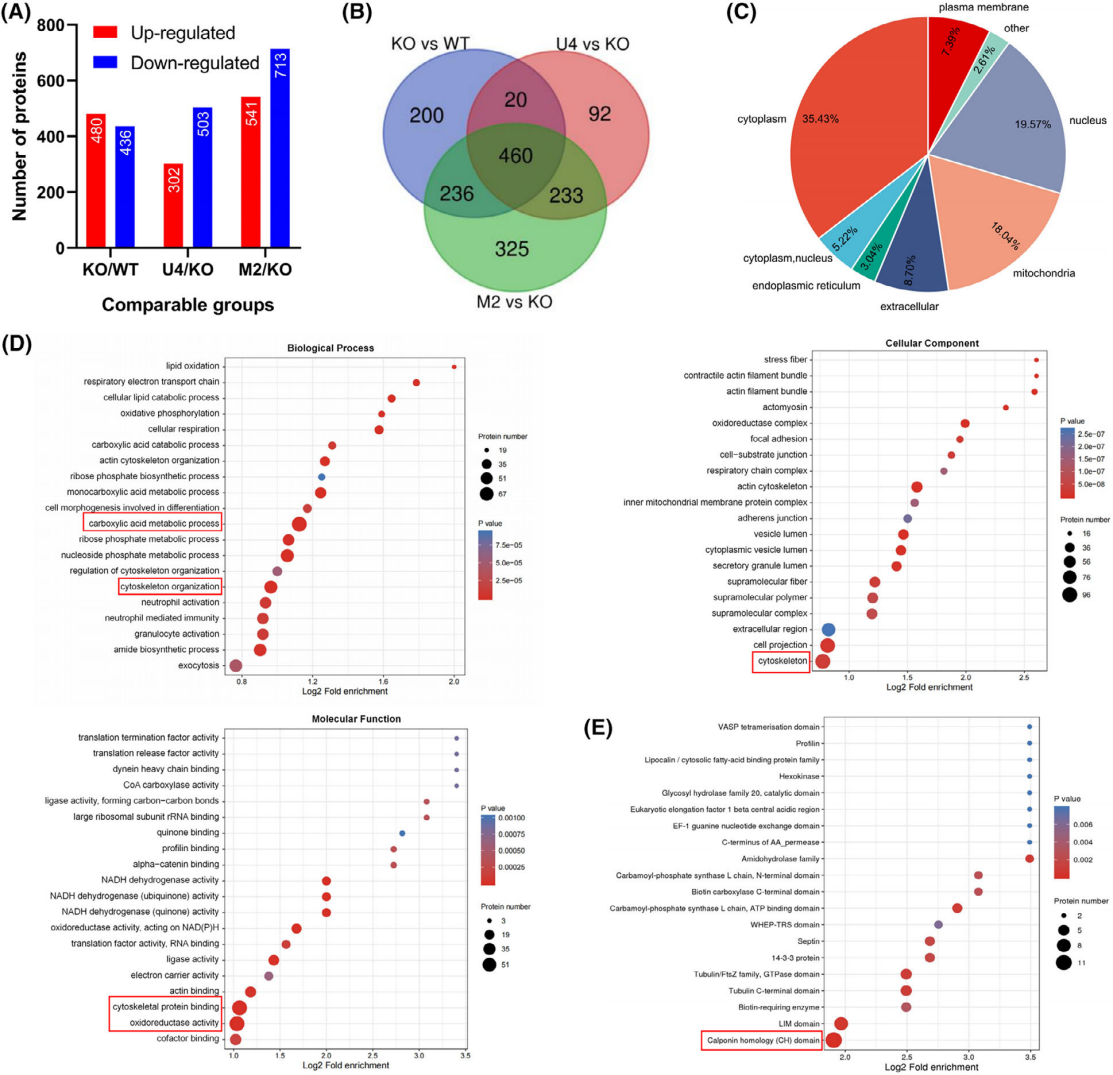
Proteomic analysis of the UCP4/MFN2 overexpression groups of MFN2‐KO cells. (A) Column chart showing the distribution of up‐ and downregulated proteins identified from the comparisons of the KO vs. WT, U4 vs. KO, and M2 vs. KO groups. (B) Venn diagram demonstrating the overlap between the KO vs. WT (blue), U4 vs. KO (pink), and M2 vs. KO (green) groups. (C) Pie chart showing the subcellular localization of the 460 overlapping proteins in (B). (D) GO enrichment analysis of the 460 overlapping proteins based on the biological process, cellular component, and molecular function categories. (E) Domain enrichment analysis of the 460 overlapping proteins. WT, A549 wild‐type cells; KO, MFN2‐KO A549 cells; U4, MFN2‐KO + UCP4‐HA A549 cells; M2, MFN2‐KO + MFN2‐FLAG A549 cells. The red boxes indicate statistically significant enrichment.

To explore common molecular mechanisms underlying the functions of MFN2 and UCP4, a TMT quantitative proteomics was conducted. A total of 5230 proteins were identified, and 4676 of these were quantified. The MFN2 wild‐type (WT), MFN2‐KO (KO), MFN2‐KO + UCP4‐HA (U4), and MFN2‐KO + MFN2‐FLAG (M2) groups were matched to form three pairs, and proteins with *P* values lower than 0.05 and fold changes in expression greater than 1.3 were considered differentially expressed proteins. We identified 916 differentially expressed proteins from the comparison of the KO versus WT groups (480 upregulated and 436 downregulated), 805 differentially expressed proteins in the comparison of the U4 versus KO groups (302 upregulated and 503 downregulated) and 1254 differentially expressed proteins from the comparison of the M2 versus KO groups (541 upregulated and 713 downregulated; Fig. 4A). Ultimately, we confirmed 460 overlapping genes (280 upregulated and 180 downregulated) among the three groups using a Venn diagram (Fig. 4B).

The mass spectrometry results revealed that the 460 overlapping proteins were localized mainly in the cytoplasm (35.43%), nucleus (19.57%), and mitochondria (18.04%; Fig. 4C). Subsequently, we conducted functional classification and enrichment analyses of the abovementioned overlapping proteins. The Gene Ontology (GO) functional classification aims to determine the composition of proteins or genes at a particular functional level and comprises three categories: biological process, molecular function, and cellular component [30]. In these three categories, the highest numbers of proteins were annotated with the terms cellular process (404 proteins), cell (441 proteins)/intracellular (431 proteins), and binding (310 proteins) contained the most proteins (Fig. S2A). In addition, a GO enrichment analysis showed that several GO terms, including ‘carboxylic acid metabolic process’, ‘cytoskeleton organization’, ‘cytoskeleton’, ‘cytoskeletal protein binding’, and ‘oxidoreductase activity’, were significantly enriched (Fig. 4D). Cluster of Orthologous Groups (COG) is a database used to classify orthologous gene products [31]. Among the 24 COG functional categories, the cluster for cytoskeleton (64 proteins) was prominently represented, followed by energy production and conversion (62 proteins; Fig. S2B). Protein domains refer to conserved repetitive regions (consisting of 25–500 amino acid residues) with a specific structure and independent function in different proteins. Notably, a protein domain enrichment analysis showed that 11 calponin homology (CH) domains, which are needed for cytoskeletal dynamics and calcium mobilization [32], were significantly enriched in the 460 overlapping proteins (Fig. 4E). Overall, these findings indicated that MFN2 and UCP4 mainly coregulate cytoskeletal dynamics and energy production, which are closely associated with the modulation of ATP and calcium homeostasis, in LUAD [33, 34].

### Protein interaction network of MFN2/UCP4 in the regulation of calcium homeostasis

**Fig. 5:**
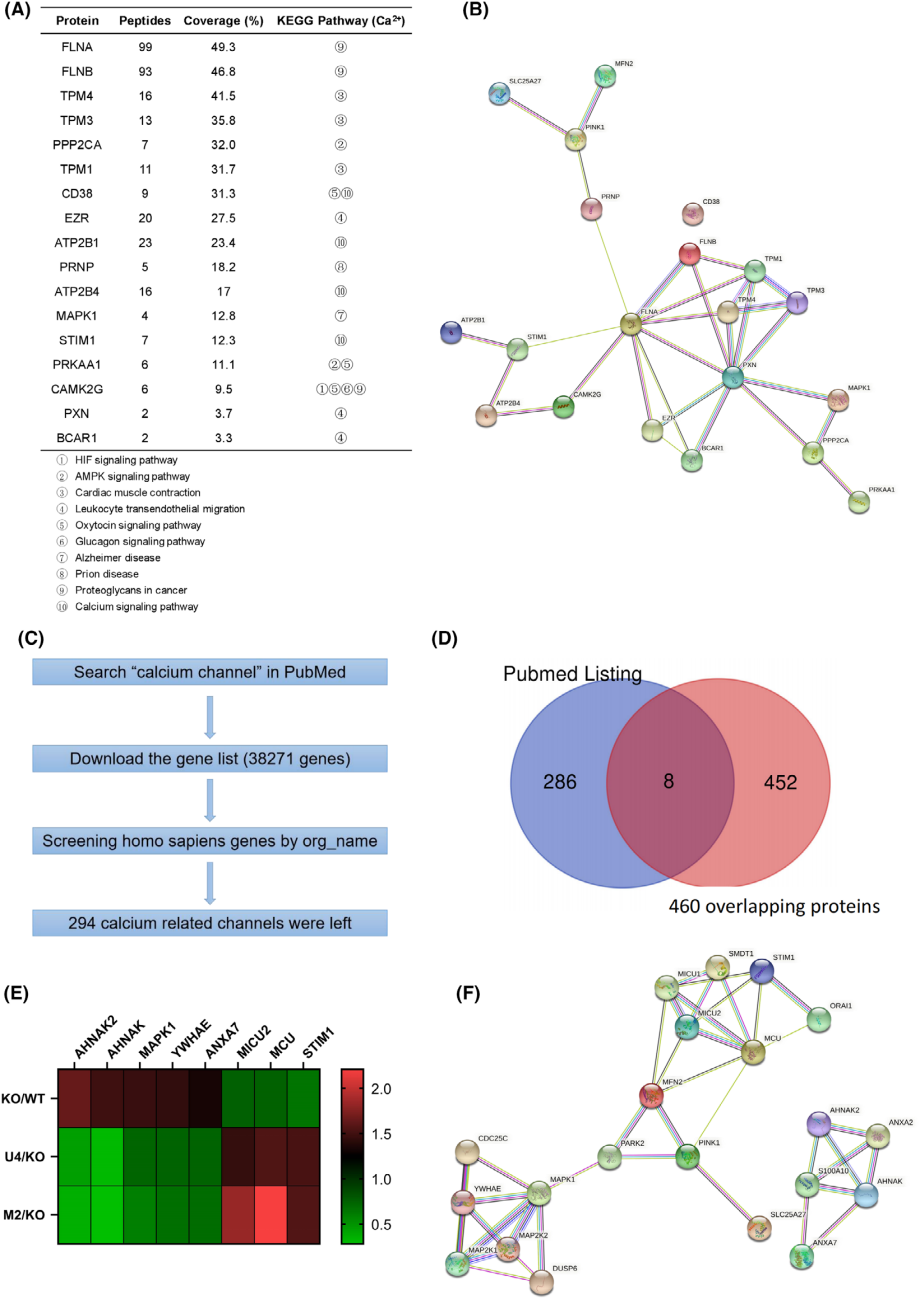
Protein–protein interaction network of MFN2/UCP4 in the regulation of calcium channels. (A) Ca^2+^‐related proteins KEGG pathway enrichment analyses. (B) PPI network of the proteins in A based on STRING. (C) Flowchart for screening calcium channels. (D) Venn diagram showing the overlap between the 460 overlapping proteins (pink) and calcium channels in PubMed (blue). (E) Heatmap of the eight overlapping genes. (F) PPI network of the proteins in (E) based on STRING.

To identify molecules and pathways involved in calcium homeostasis regulated by MFN2/UCP4 in LUAD, we further performed a KEGG pathway enrichment analysis of the 460 overlapping proteins using the DAVID database [35]. The analysis showed that signaling pathways involved in thermogenesis, nonalcoholic fatty liver disease (NAFLD), and oxidative phosphorylation were enriched (Fig. S2C). Notably, the KEGG pathway analysis of the 280 upregulated overlapping genes found that the calcium signaling pathway was enriched (Fig. S3A). We also screened a total of 17 genes (belonging to 10 different pathways) correlated with Ca^2+^ regulation from 51 KEGG pathway enrichment maps (Fig. 5A and Fig. S3B). Among these 17 genes, CD38, FLNB, TPM1, and FLNA were among the top 50 differentially expressed genes (DEGs; Fig. S2D). Moreover, the possible PPI network of these proteins was constructed using STRING software (Fig. 5B). In particular, PTEN‐induced putative kinase 1 (PINK1), which is linked to Parkinson's disease, mitochondrial homeostasis, the Warburg effect in cancer, the cell cycle, autophagy, and inflammatory responses [36], was identified as a key node connecting MFN2/UCP4 and other nodes in the regulation of calcium homeostasis.

We subsequently used an alternative screening method to verify the relationship between MFN2/UCP4 and calcium channels in LUAD, as described previously [26]. The screening flowchart is shown in Fig. 5C. We ultimately obtained 38 271 genes from a keyword search (calcium channel) of PubMed, and 294 of these genes were encoded in the *Homo sapiens* genome. The calcium channel‐related genes in humans (*n* = 294) were then intersected with the 460 overlapping genes to obtain eight intersecting genes (Fig. 5D): five upregulated (AHNAK2, AHNAK, MAPK1, YWHAE, and ANXA7) and three downregulated (MICU2, MCU, and STIM1) genes. The expression levels of the eight intersected genes were visualized in a heatmap (Fig. 5E). Additionally, a gene set enrichment analysis (GSEA) revealed that MITOCHONDRIAL CALCIUM ION TRANSPORT was significantly enriched (MFN2_down: NES = −3.167, FDR = 0.015; UCP4_up: NES = 2.116, FDR = 0.031; MFN2_up: NES = 2.405, FDR = 0.016; Fig. S4). Because the mitochondrial uniporter complex (containing MCU, MICU1/2/3, MCUb, MCUR1, and EMRE) is an essential regulator of mitochondrial Ca^2+^ uptake and export [37, 38], we sought to construct the PPI of MFN2/UCP4 in the control of mitochondrial Ca^2+^ homeostasis with STRING. PINK1 may play a crucial role in MFN2/UCP4‐mediated mitochondrial calcium homeostasis (Fig. 5F), consistent with the results shown in Fig. 5B. Collectively, these results indicate that MFN2/UCP4 may participate in regulating calcium homeostasis in LUAD by regulating the PINK1‐associated PPI network.

### PINK1 increases the MFN2/UCP4‐mediated intracellular Ca^2+^ concentration

**Fig. 6:**
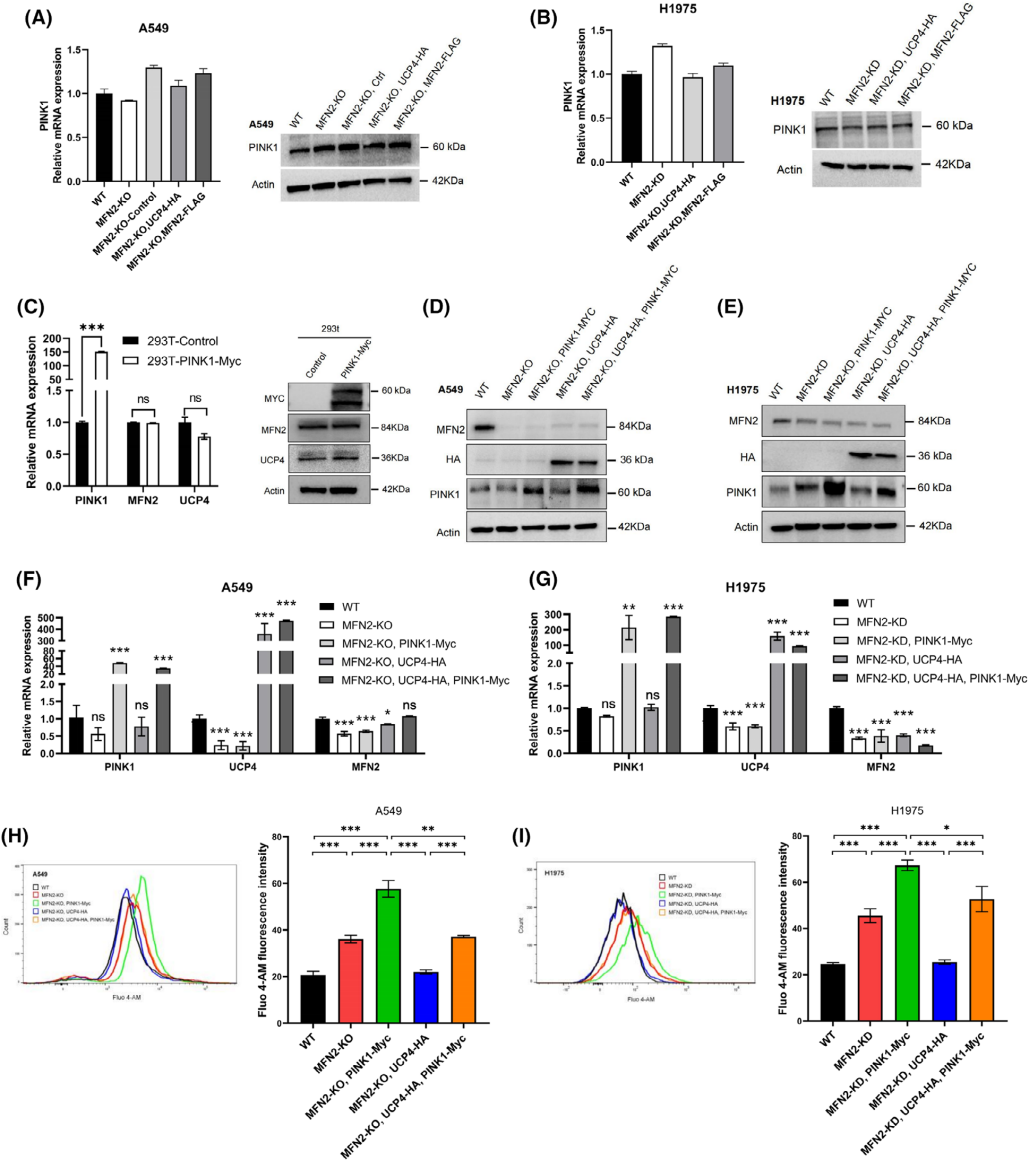
PINK1 increases the MFN2/UCP4‐mediated intracellular calcium concentration. (A, B) qRT–PCR and western blot analysis of PINK1 expression in MFN2‐KO A549 (A) and MFN2‐KD H1975 (B) cells. (C) qRT–PCR and western blot analysis of UCP4, MFN2, and PINK1 expression in 293T cells transduced with PINK1‐Myc. (D, E) Western blot analysis of UCP4, MFN2, and PINK1 expression in MFN2‐KO A549 (D) and MFN2‐KD H1975 (E) cells stably transfected with UCP4‐HA or PINK1‐Myc. (F, G) qRT–PCR analysis of UCP4, MFN2, and PINK1 expression in MFN2‐KO A549 (F) and MFN2‐KD H1975 (G) cells stably transfected with UCP4‐HA or PINK1‐Myc. (H, I) The Ca^2+^ levels in MFN2‐KO A549 (H) and MFN2‐KD H1975 (I) cells were quantified by flow cytometry (histograms on the left). The mean Fluo‐4 AM fluorescence intensity is shown on the right. *P < 0.05; **P < 0.01; ***P < 0.001; n.s., nonsignificant (Student's *t*‐test). Values were mean ± SEM of three independent experiments.

To confirm the role of the PINK1‐associated PPI network in MFN2/UCP4‐mediated calcium homeostasis, we first examined the expression of PINK1 in A549 and H1975 cells and showed that the mRNA and protein expression levels of PINK1 itself did not change significantly after MFN2 deletion or UCP4/MFN2 overexpression (Fig. 6A,B). We then transiently transfected PINK1‐Myc into 293T cells and found that the overexpression of PINK1 also did not affect the expression of MFN2 and UCP4 (Fig. 6C), which suggested that the regulation of MFN2/UCP4, in which PINK1 is involved, is not regulated through their expression. We ultimately achieved overexpression of PINK1‐Myc by lentiviral packaging based on MFN2‐KO A549 or MFN2‐KD H1975 cells, respectively. The overexpression efficiency of PINK1 was verified by RT–PCR and western blotting (Fig. 6D–G). Remarkably, lentiviral vector‐mediated PINK1 overexpression not only further increased the intracellular calcium concentration induced by MFN2 deficiency but also increased the intracellular calcium concentration restored by UCP4 overexpression in A549 and H1975 cells (Fig. 6H,I). These findings fully demonstrate that PINK1 is indeed involved in the MFN2/UCP4‐mediated regulation of calcium homeostasis and is independent of its expression regulation, and the detailed mechanism deserves further investigation.

### Low levels of MFN2/UCP4 are correlated with poor prognosis in LUAD patients

**Fig. 7:**
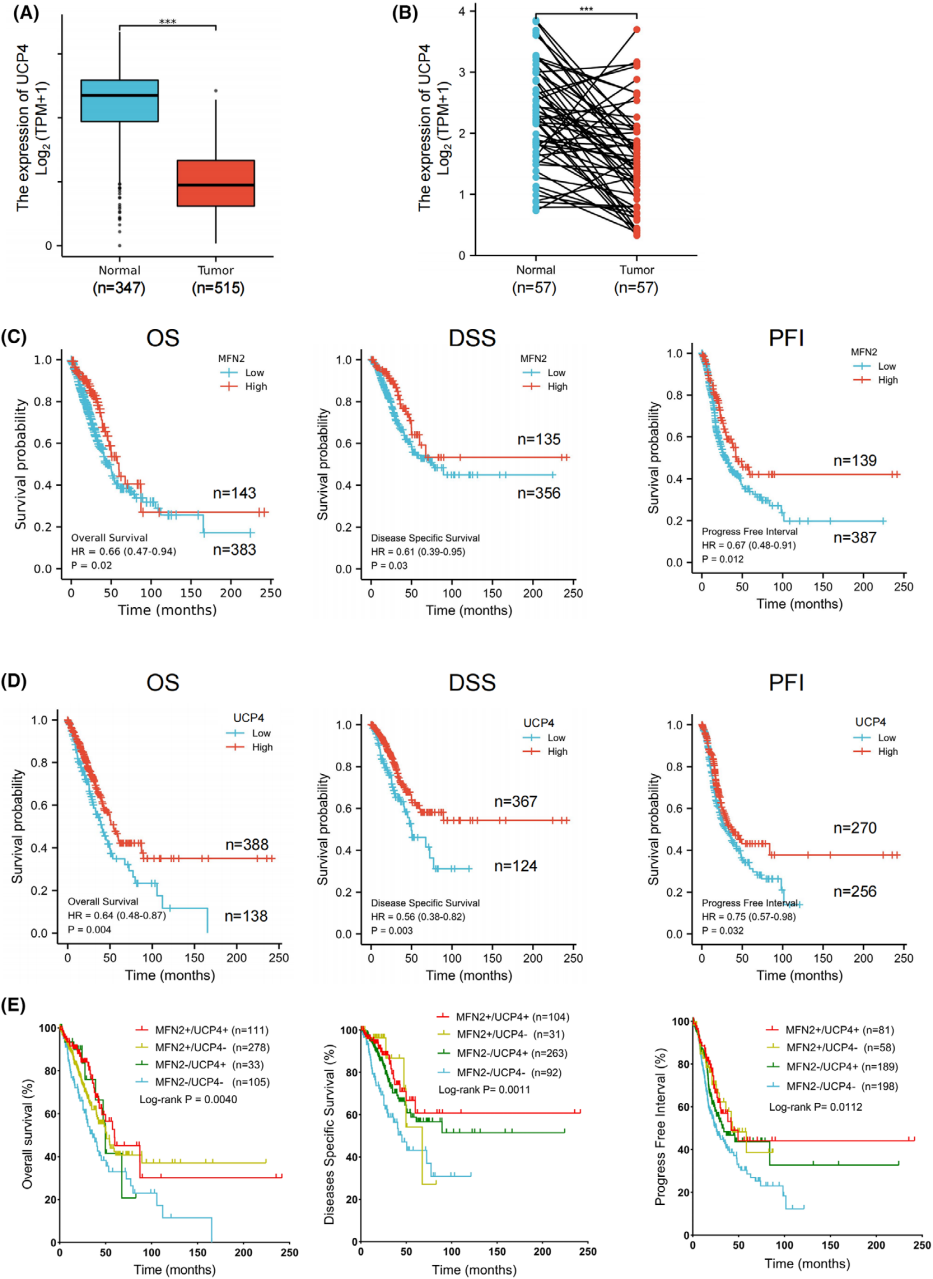
Downregulation of MFN2/UCP4 expression in LUAD patients is correlated with poor prognosis. (A, B) Relative UCP4 mRNA levels from the TCGA database in unpaired (A, Mann–Whitney *U* test, *n* = 347^normal^/515^tumor^) and paired (B, Student's *t*‐test, *n* = 57^normal^/57^tumor^) normal and LUAD tissues. (C–E) Kaplan–Meier analysis of overall survival according to MFN2 (C, OS: *n* = 143^high^/383^low^; DSS: *n* = 135^high^/356^low^; PFI: *n* = 139^high^/387^low^) or UCP4 (D, OS: *n* = 388^high^/138^low^; DSS: *n* = 367^high^/124^low^; PFI: *n* = 270^high^/256^low^) expression alone as well as coexpression (E) in LUAD patients from the TCGA database. The log‐rank test was used to determine *P* values for (C–E).

Calcium is necessary for cells to execute multiple physiological functions [39]. Dysregulation of calcium homeostasis in cancer not only leads to abnormal tumor growth and metastasis but also aids the evasion of cell death, escape from immune surveillance and drug resistance, resulting in poor prognosis [39, 40]. We then investigated whether the dysregulation of calcium homeostasis mediated by aberrant expression of MFN2 and UCP4 is related to the clinical prognosis of LUAD patients. As shown in Fig. 1A,B, MFN2 was expressed at low levels in LUAD cells. Based on the same database analysis, UCP4 expression was also significantly reduced in unpaired (Fig. 7A) and paired (Fig. 7B) LUAD samples. Moreover, survival was evaluated by the Kaplan–Meier method. A MFN2^Low^ status predicted shorter overall survival (OS) and disease‐specific survival (DSS) times and a shorter progression‐free interval (PFI; Fig. 7C). Similarly, reduced UCP4 expression was also correlated with poorer OS, DSS, and PFI in patients with LUAD (Fig. 7D). More importantly, low MFN2 and UCP4 coexpression predicted a worse prognosis in LUAD patients than high coexpression (Fig. 7E). These results suggest that MFN2/UCP4 act as tumor suppressors in LUAD and are potential prognostic markers for lung cancer.

## Discussion

Mitochondria, important organelles in human tissue cells, are the primary site of energy metabolism and ROS generation [41]. Mitochondrial dysfunction is strongly associated with the development of many diseases, including cancer, aging, diabetic kidney disease, cardiovascular disease, and neurodegenerative disease [41]. In cancer, mitochondrial dysfunction is considered beneficial because it can weaken cancer cells. Therefore, investigations of the role of mitochondrial dysfunction in lung cancer are crucial for designing future mitochondria‐targeted therapeutic strategies for lung cancer.

We initially established an A549 cell line with the complete KO of MFN2. First, A549 is one of the most commonly used LUAD cell lines in research. Second, the expression of MFN2 was found to be highest in the A549 lung adenocarcinoma cell line. In particular, due to their low expression of MFN2, we initially assumed that PC9 and H1975 cells may not be suitable for generating KO cell lines. Third, A549 cells had the lowest intracellular Ca^2+^ concentration among LUAD cells under the same staining conditions; thus, A549 cells were selected considering the expected significance of the results. Fourth, in the process of establishing the MFN2 gene‐KO cell line, we screened more than 150 monoclonal cells, and the final sequencing results showed that only clone MFN2#‐25 in the third screening experiment exhibited simultaneous KO of the MFN2 allele on both chromosomes. The screening workload is very heavy. We also investigated the mitochondrial dysfunction caused by MFN2 deletion using siRNA and found that the results from H1975 cells were more significant and consistent (Fig. S1A–H). Thus, the A549 and H1975 cell lines were selected for further investigation.

The data further demonstrated that UCP4 gene expression was decreased following MFN2 KO (Fig. 1D–H), indicating a direct relationship between the expression of both proteins. We attempted to explore how MFN2 regulates UCP4 directly or indirectly. MFN2 is localized on the outer mitochondrial membrane, whereas UCP4 resides on the inner mitochondrial membrane [17]. We thus speculated the potential existence of a direct interaction between MFN2 and UCP4. However, no direct binding of MFN2 to UCP4 was observed by CO‐IP assay (Fig. S5A). To investigate whether MFN2 directly regulates the promoter activity of UCP4, we constructed a promoter region ~ 2000 bp upstream of the UCP4 start site [42]. The luciferase reporter gene assay revealed no significant regulation of the UCP4 promoter by MFN2 (Fig. S5B). Studies have shown that NF‐κB can directly regulate UCP4 gene transcription by binding to its promoter region [42]. Paradoxically, MFN2 deficiency could activate the insulin signaling molecule NF‐κB [43]. However, NF‐κB activation is involved in MFN‐2 degradation caused by calpain upregulation [44]. In summary, MFN2 may regulate UCP4 through negative feedback regulation with NF‐κB, and the exact mechanism still requires further study.

The similar mitochondrial localization of MFN2 and UCP4 suggests possible overlap in the regulation of specific functions. The results from a KEGG pathway enrichment analysis confirmed our speculation (Figs S2C and S3B). MFN2 and UCP4 play a coregulatory role in numerous disorders, including nonalcoholic fatty liver disease (NAFLD), Parkinson's disease, Alzheimer's disease (AD), and Huntington's disease. Evidence indicates potential correlations between the MFN2/UCP4 genes and AD, a progressive neurodegenerative disease [45, 46]. Mitochondrial dysfunction is a common factor driving these diseases, and the influence of intracellular calcium homeostasis on mitochondrial dysfunction cannot be ignored. Moreover, cytoskeleton‐related entities were identified as significantly enriched in the GO, COG, and KEGG analyses (Figs 4D, S2B and S3B). Moreover, our protein domain enrichment analysis showed that the calponin homology (CH) domain, which plays an important role in cytoskeletal dynamics and calcium mobilization [32], was enriched (Fig. 4E). Many studies have revealed that the actin cytoskeleton is not only a modulator of mitochondrial structure and function [47, 48] but also an upstream and downstream regulator of Ca^2+^ signaling [49, 50]. This observation is consistent with our results, indicating that MFN2 and UCP4 can coregulate intracellular calcium homeostasis in A549 cells (Fig. 2G). Our findings thus delineate the potential contributions of MFN2 and UCP4 to multiple diseases and particularly to the regulation of calcium homeostasis in A549 lung cancer cells.

Currently, the common mechanism of MFN2 and UCP4 has not been reported. To further explore the underlying molecular mechanisms of MFN2 and UCP4 in A549 cells, we used two different methods to predict the possible molecular pathways (Fig. 5) and found that PINK1 may play a role in controlling MFN2/UCP4‐mediated calcium homeostasis in A549 cells. Our data confirm that PINK1 overexpression increased the intracellular calcium concentrations in MFN2‐deficient cells and also increased the intracellular calcium concentrations restored by UCP4 overexpression (Fig. 6H,I). However, elevated expression of PINK1 had little effect on the expression of MFN2 and UCP4, suggesting that the regulation of MFN2/UCP4 by PINK1 is mediated through other pathways. PINK1 (PARK6), a mitochondrial serine/threonine kinase, plays a critical role in modulating mitophagy through the PINK1‐MFN2‐PARKIN (PARK2) pathway [51]. Interestingly, in addition to their roles in autophagy, PINK1 and Parkin are essential for the regulation of Ca^2+^ homeostasis and ER–mitochondria tethering [52]. MFN2 is another major mediator of ER–mitochondria tethering, which is associated with numerous physiological processes, such as autophagy, Ca^2+^ homeostasis, inflammation, lipid transfer, and cell death [53]. Collectively, our findings provide information helpful for clarifying the coregulatory mechanism of MFN2 and UCP4, which may regulate intercellular calcium homeostasis through PINK1‐mediated mitophagy or ER–mitochondria tethering and thereby affect the biological properties of lung cancer cells. Moreover, recent studies have reported that CACNA1E is a functional calcium channel and that its mRNA and protein levels are elevated in non‐small‐cell lung cancer [54]. MFN2/UCP4 may also regulate the intracellular calcium concentration via CACNA1E or other calcium channels. In conclusion, more extensive research is needed to determine the molecular mechanisms underlying the regulatory roles of MFN2 and UCP4 in LUAD.

The study revealed the possible role of UCP4 in lung cancer. Previous studies have shown that UCP4 localizes to the outer mitochondrial membrane [17] and is widely expressed in neuronal cells and neurosensory cells [13, 14]. How and why is this mitochondrial protein essential for the development of lung cancer? After the investigation of UCP4 expression in tissues using the Human Protein Atlas website, we found that UCP4 is widely expressed in various human tissues and exhibits low tissue specificity (Fig. S6A). In line with previous reports, RNA single‐cell type expression indicated that UCP4 was enhanced in oligodendrocyte precursor cells, oligodendrocytes, excitatory neurons, inhibitory neurons, and astrocyte neuronal cells (Fig. S6B). Moreover, single‐cell results showed that UCP4 was highly expressed in glandular epithelial cells (including respiratory ciliated cells and club cells), specialized epithelial cells (including alveolar cells type 1 and 2), and endothelial cells of the lung (Fig. S6C). It is worth emphasizing that LUAD is usually a malignant tumor arising from bronchial epithelial cells or alveolar cells. Furthermore, Kaplan–Meier survival analysis revealed that lower UCP4 expression led to poor prognosis in LUAD patients (Fig. 7D), consistent with the result that UCP4 expression was generally very low in various tumor cells (Fig. S6D). In conclusion, these findings indicate a potential role of UCP4 in lung cancer development.

However, our results were derived only from a database analysis and need to be verified with clinical samples. Previous studies have shown that low expression of MFN2 is related to poor progression in hepatocellular carcinoma, lung cancer, and breast cancer [11, 55], consistent with our results. Other reports have indicated that high expression of MFN2 is linked to poor prognosis in cervical cancer [56], contradictory to our findings. The abovementioned results imply that the role of MFN2 in carcinogenesis depends on the cancer type. This study identifies MFN2 and UCP4 as potential prognostic markers and therapeutic targets in LUAD.

Overall, our study indicates a strong underlying correlation between MFN2 and UCP4 in the modulation of PINK1‐mediated calcium homeostasis. This finding allows further investigation of the synergistic function and mechanism of MFN2 and UCP4 in LUAD. Moreover, we found that MFN2 and UCP4 were expressed at low levels in LUAD clinical samples and that their low expression was significantly associated with poor prognosis. These data suggest that MFN2 and UCP4 may be novel therapeutic targets in lung cancer and may provide new insights into the clinical management of lung cancer.

## Conflict of interest

The authors declare no conflict of interest.

## Author contributions

JZ, QW, NL, and SZ designed the study; JZ, QW, and LP performed the experiments; JZ, QW, LP, YZ, and WW analyzed and interpreted the data; QZ, NL, and QW revised the manuscript; JZ and QZ wrote the manuscript; QW and SZ decided to submit the article for publication.

## Supporting information


**Fig. S1.** MFN2 deficiency causes mitochondrial dysfunction in lung adenocarcinoma cells.

**Fig. S2.** Proteomic analysis of the UCP4/MFN2 overexpression groups of MFN2‐KO cells.

**Fig. S3.** Screening of calcium‐related KEGG pathways.

**Fig. S4.** GSEA of MFN2/UCP4 in the regulation of mitochondrial calcium ion transport.

**Fig. S5.** MFN2 cannot directly regulate UCP4 expression.

**Fig. S6.** Expression of UCP4 in the lung.

**Table S1.** Primers used in this study.
**Table S2.** Detailed gene lists for the mitochondrial PCR array and mitochondrial energy metabolism PCR array.

## Data Availability

All data supporting the results of this study are available from the corresponding authors upon reasonable request.
