# Supplementary figures and images for: MFN2 deficiency affects calcium homeostasis in lung adenocarcinoma cells via downregulation of UCP4
Source: FEBS Open Bio. 2023 Mar 14;13(6):1107–24. doi: 10.1002/2211-5463.13591 (PMC10240348; doi:10.1002/2211-5463.13591)

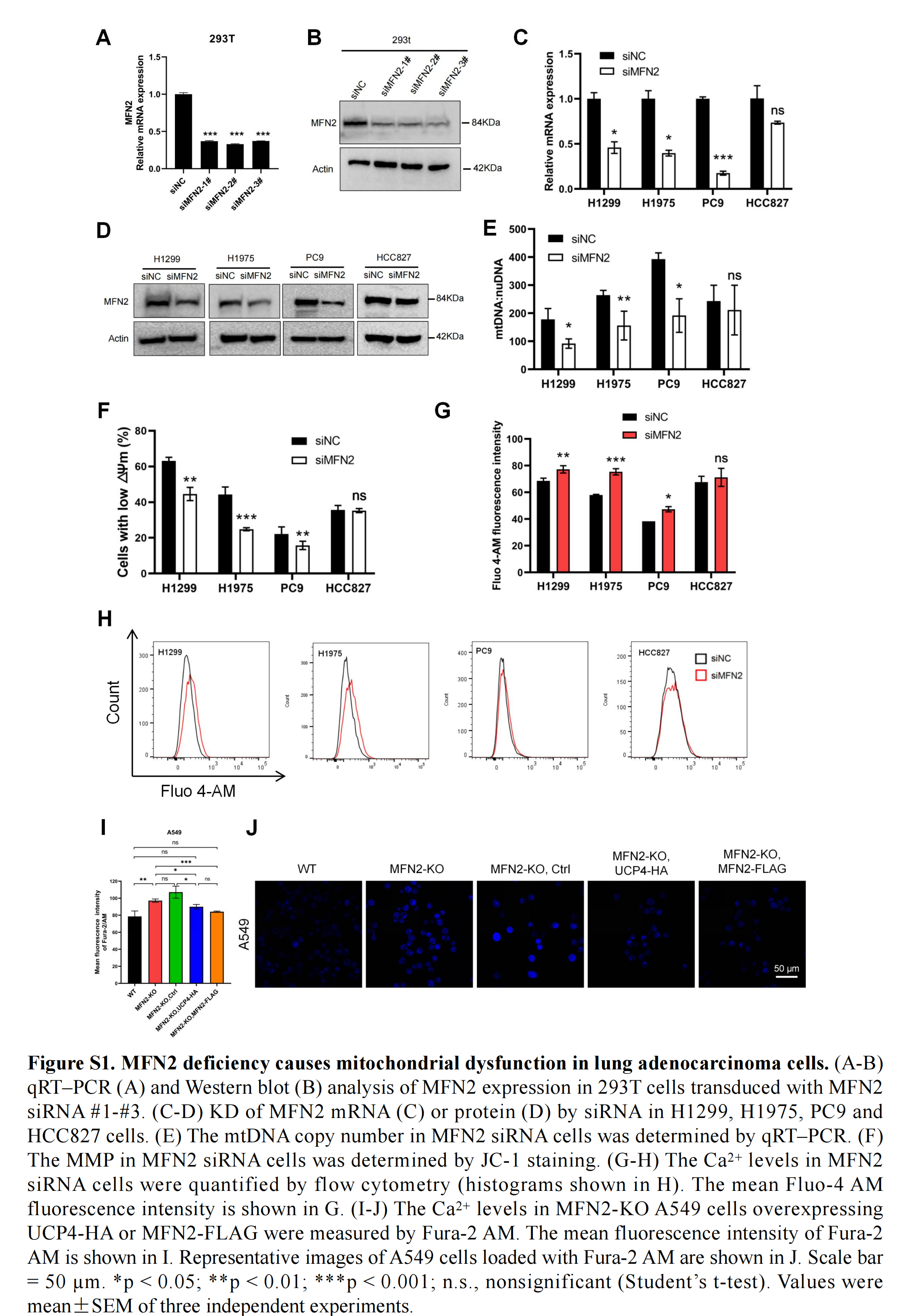

Supplement: Supplementary file 1 — Fig. S1. MFN2 deficiency causes mitochondrial dysfunction in lung adenocarcinoma cells. [file FEB4-13-1107-s003.tif]

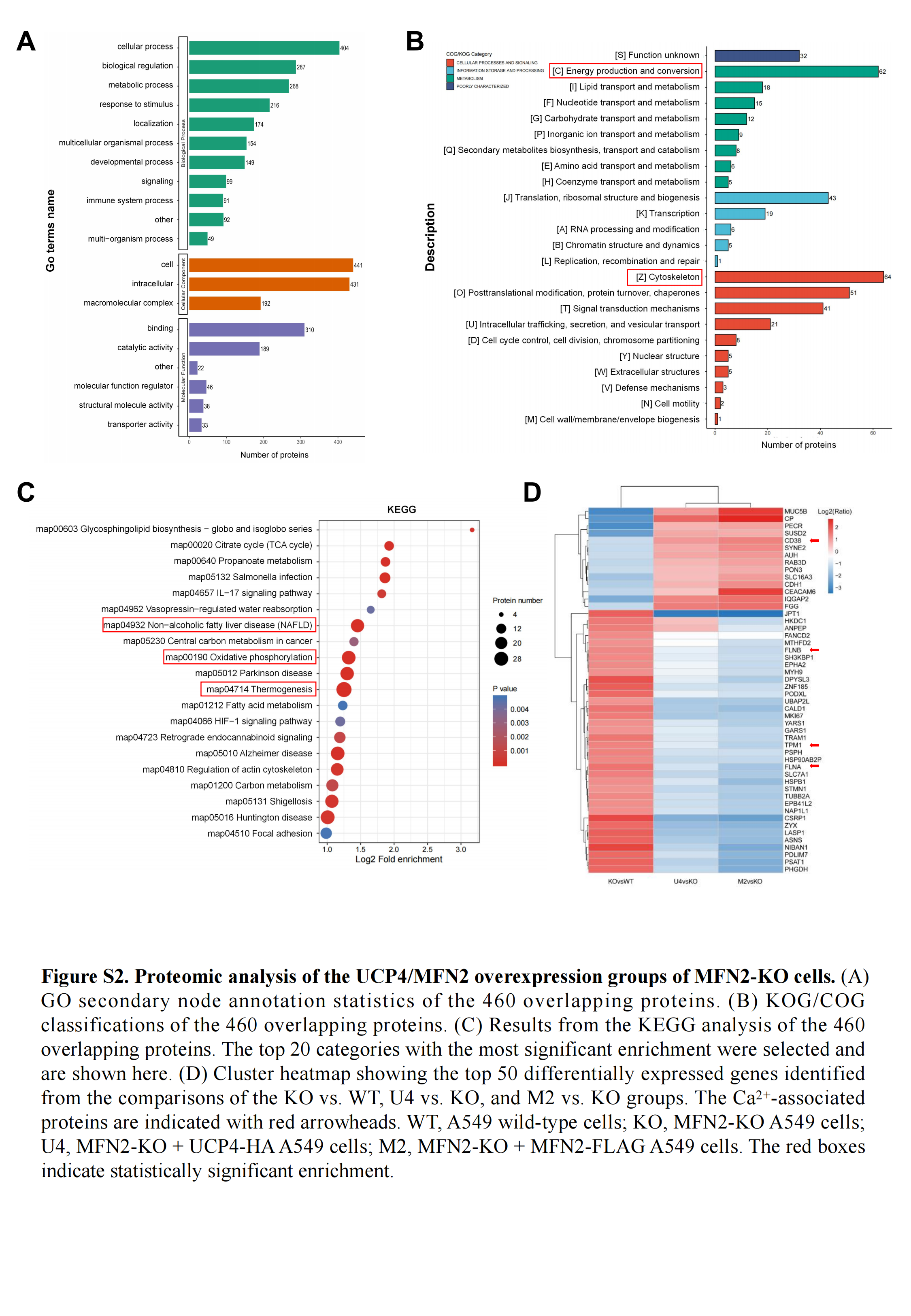

Supplement: Supplementary file 2 — Fig. S2. Proteomic analysis of the UCP4/MFN2 overexpression groups of MFN2‐KO cells. [file FEB4-13-1107-s002.tif]

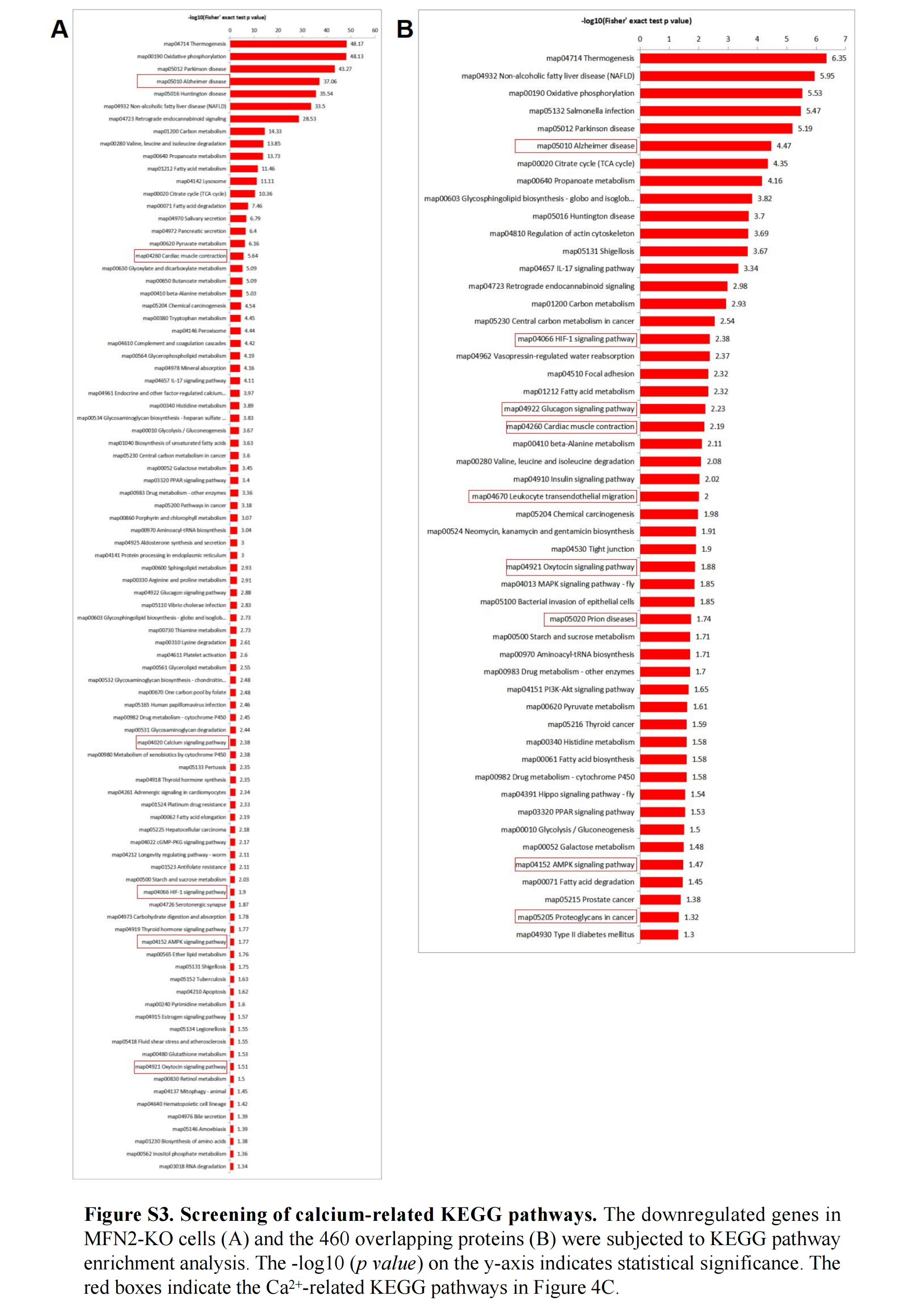

Supplement: Supplementary file 3 — Fig. S3. Screening of calcium‐related KEGG pathways. [file FEB4-13-1107-s006.tif]

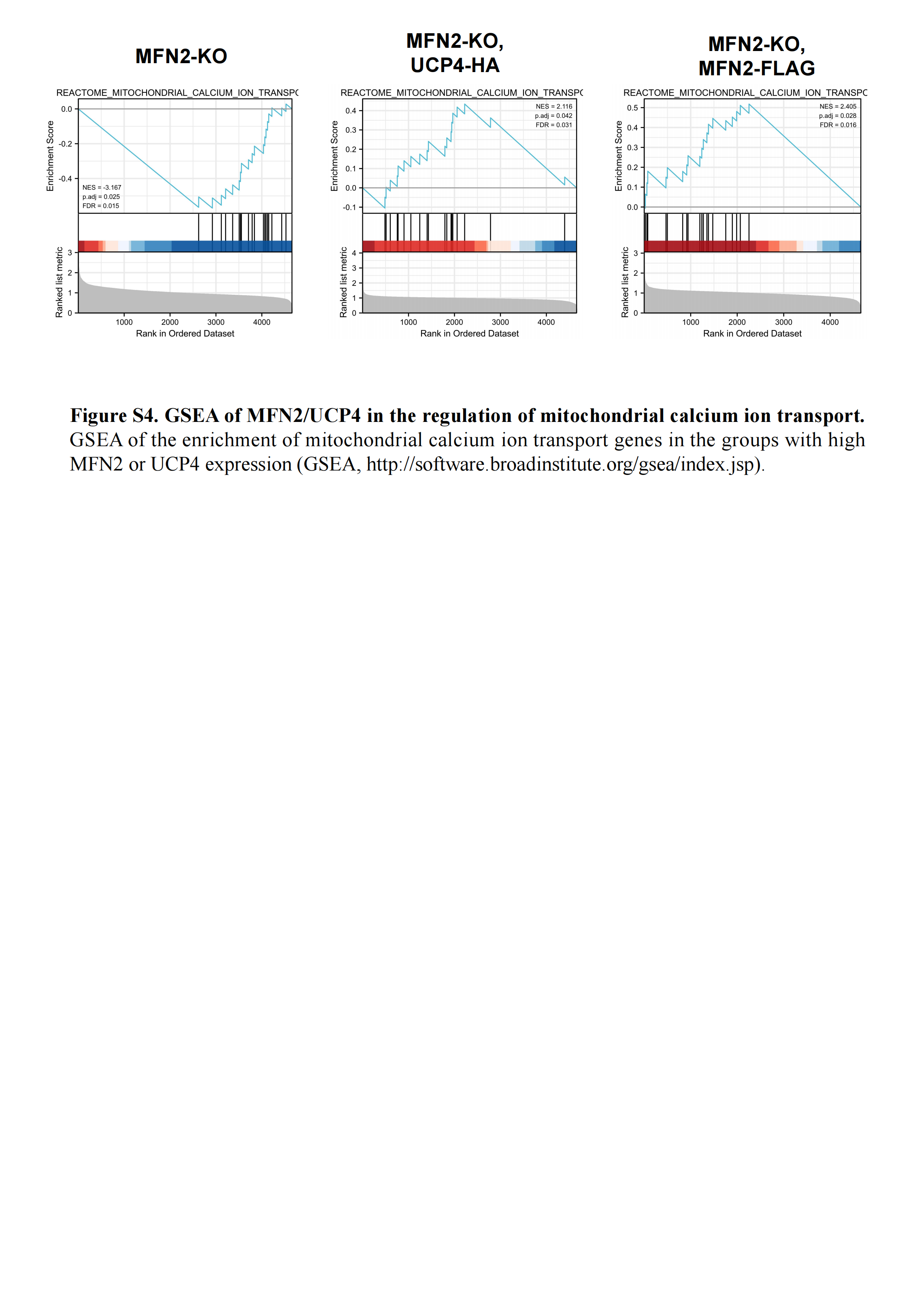

Supplement: Supplementary file 4 — Fig. S4. GSEA of MFN2/UCP4 in the regulation of mitochondrial calcium ion transport. [file FEB4-13-1107-s005.tif]

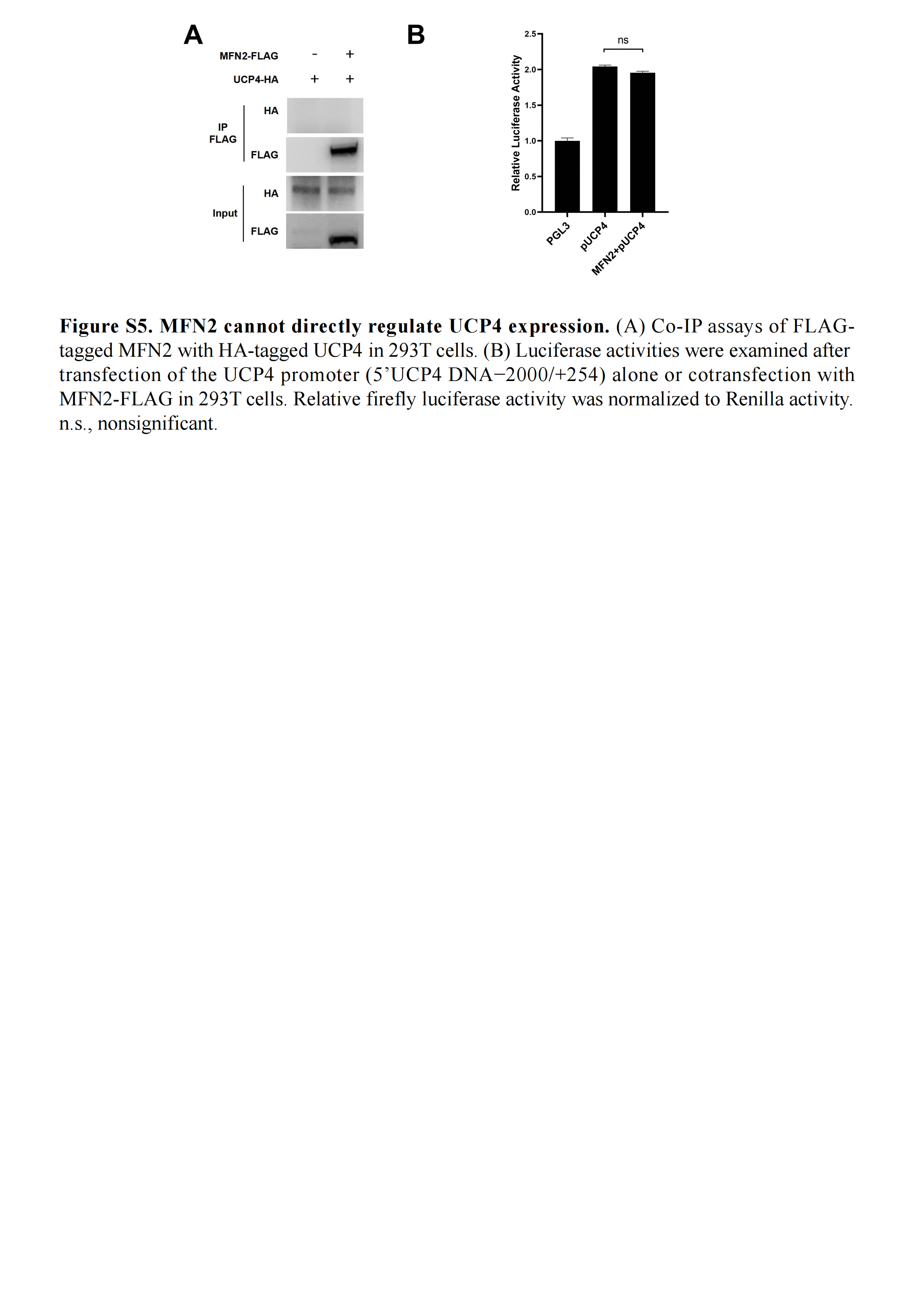

Supplement: Supplementary file 5 — Fig. S5. MFN2 cannot directly regulate UCP4 expression. [file FEB4-13-1107-s001.tif]

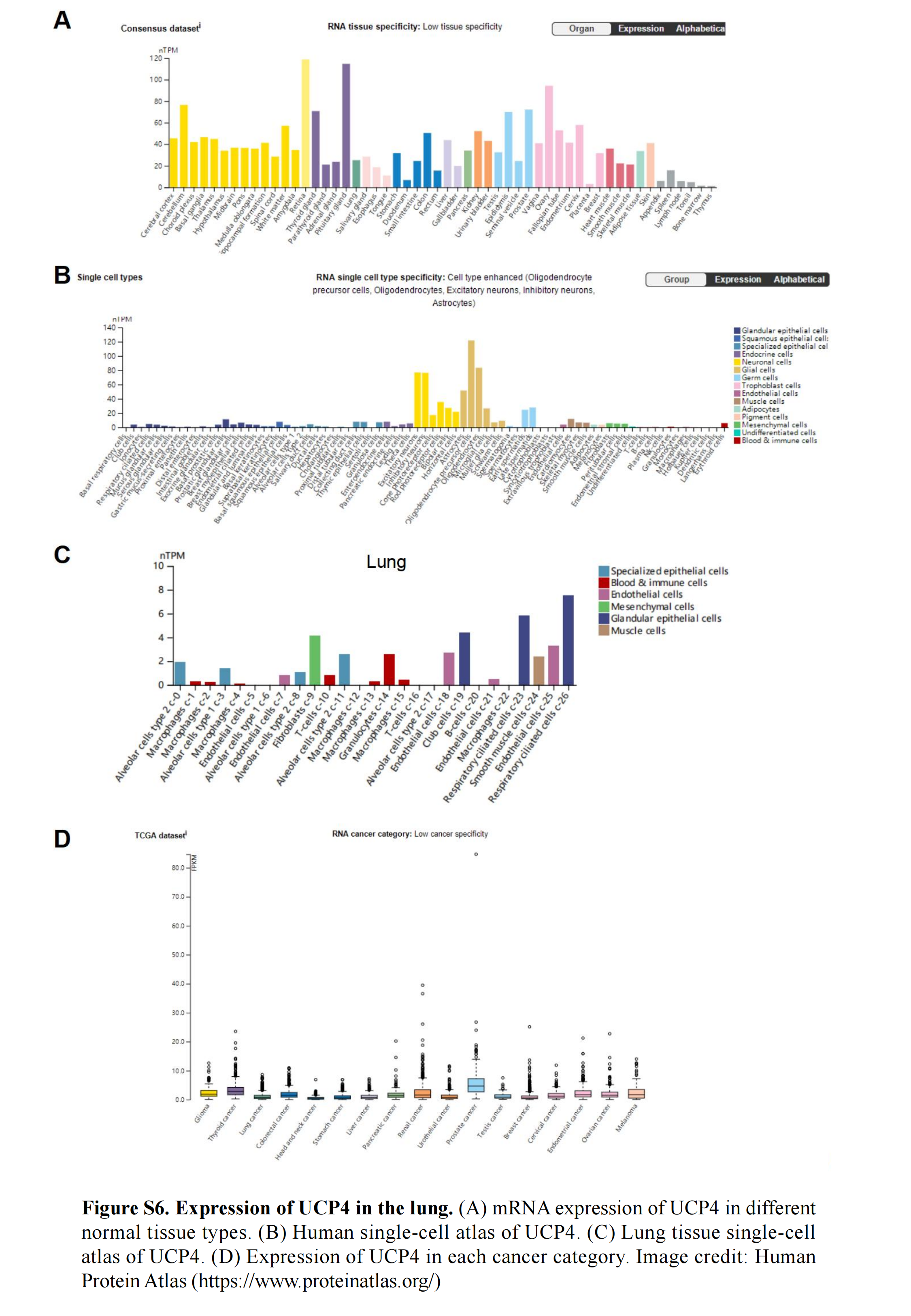

Supplement: Supplementary file 6 — Fig. S6. Expression of UCP4 in the lung. [file FEB4-13-1107-s004.tif]
