# Supplementary material for: MFN2 deficiency affects calcium homeostasis in lung adenocarcinoma cells via downregulation of UCP4
Source: FEBS Open Bio. 2023 Mar 14;13(6):1107–24. doi: 10.1002/2211-5463.13591 (PMC10240348; doi:10.1002/2211-5463.13591)
Supplement: Supplementary file 7 — Table S1. Primers used in this study. Table S2. Detailed gene lists for the mitochondrial PCR array and mitochondrial energy metabolism PCR array. [file FEB4-13-1107-s007.docx]

Supplementary Table 1: Primers used in this study

| **Primers for quantitative real-time PCR** | |
| --- | --- |
| β-actin-For | GGCTGTATTCCCCTCCATCG |
| β-actin-Rev | CCAGTTGGTAACAATGCCATGT |
| Hu-CDKN2A- For | ATGGAGCCTTCGGCTGACT |
| Hu-CDKN2A-Rev | GTAACTATTCGGTGCGTTGGG |
| Hu-NOS2-For | TTCAGTATCACAACCTCAGCAAG |
| Hu-NOS2-Rev | TGGACCTGCAAGTTAAAATCCC |
| Hu-SLC25A27-For | AAAACCATTGCGATTTCGTGG |
| Hu-SLC25A27-Rev | CCCAAAGCCCTCGTATTCCT |
| Hu-COX6A2-For | ATCATGGCTTTGCCTCTGA |
| Hu-COX6A2-Rev | AGCAGACGCCAGGTACGA |
| Hu- EDN1-For | AGAGTGTGTCTACTTCTGCCA |
| Hu- EDN1-Rev | CTTCCAAGTCCATACGGAACAA |
| Hu-TNF-For | CCTCTCTCTAATCAGCCCTCTG |
| Hu-TNF-Rev | GAGGACCTGGGAGTAGATGAG |
| Hu-MFN2-For | CTCTCGATGCAACTCTATCGTC |
| Hu-MFN2-Rev | TCCTGTACGTGTCTTCAAGGAA |
| Hu-mtDNA-ND6-For | GCCTGGTGATAGCTGGTTGT |
| Hu-mtDNA-ND6-Rev | GGTGGCTGCTTTTAGGCCTA |
| Hu-nuDNA-β-Actin-For | AGCGGGAAATCGTGCGTGAC |
| Hu-nuDNA-β-Actin-Rev | AGGCAGCTCGTAGCTCTTCTC |
| Hu-PINK1-For | GGAGGAGTATCTGATAGGGCAG |
| Hu-PINK1-Rev | AACCCGGTGCTCTTTGTCAC |
| Hu-NDUFS1-For | TGCCCTGTAGGTGCCCTAA |
| Hu-NDUFS1-Rev | CCAACCGCATCCATTACATCAAT |
| Hu-NDUFV2-For | GGTTGGGGAGACTACACCTGA |
| Hu-NDUFV2-Rev | CTTGGCCCTGGTTTTGGGAT |
| Hu-SDHA-For | CAAACAGGAACCCGAGGTTTT |
| Hu-SDHA-Rev | CAGCTTGGTAACACATGCTGTAT |
| Hu-SDHD-For | CATCTCTCCACTGGACTAGCG |
| Hu-SDHD-Rev | TCCATCGCAGAGCAAGGATTC |
| Hu-UQCRC2-For | TTCAGCAATTTAGGAACCACCC |
| Hu-UQCRC2-Rev | GGTCACACTTAATTTGCCACCAA |
| Hu-UQCRH-For | GAGGACGAGCAAAAGATGCTT |
| Hu-UQCRH-Rev | CGAGAGGAATCACGCTCATCA |
| Hu-COX5B-For | ATGGCTTCAAGGTTACTTCGC |
| Hu-COX5B-Rev | CCCTTTGGGGCCAGTACATT |
| Hu-COX7A2L-For | CACCAACTAAACTGACCTCCG |
| Hu-COX7A2L-Rev | GGGCACACCATCAGCTTTCT |
| Hu-ATP5G3-For | CCAGAGTTGCATACAGACCAAT |
| Hu-ATP5G3-Rev | CCCATTAAATACCGTAGAGCCCT |
| Hu-ATP5L-For | ACTACGCCAAGGTTGAGCTG |
| Hu-ATP5L-Rev | GCCCCGCTTGCCTATAATCTC |
|  |  |
| **Oligonucleotides used in CRISPR-CAS9 system** | |
| Target 1：5′-GCTCTTCTCTCGATGCAACT-3′ | |
| Target 2：5′-CACTTAAGCACTTTGTCACT-3′ | |
| Target 3：5′-GGAGAGCGCCACCTTCCTTG-3′ | |
|  |  |
| **Oligonucleotides used for cloning sgRNA expression vector** | |
| MFN2 sgRNA1-F | CACCG GAGTTGCATCGAGAGAAGAGC |
| MFN2 sgRNA1-R | AAAC GCTCTTCTCTCGATGCAACTC |
| MFN2 sgRNA2-F | CACCG GAGTGACAAAGTGCTTAAGTG |
| MFN2 sgRNA2-R | AAAC CACTTAAGCACTTTGTCACTC |
| MFN2 sgRNA3-F | CACCG GCAAGGAAGGTGGCGCTCTCC |
| MFN2 sgRNA3-R | AAAC GGAGAGCGCCACCTTCCTTGC |
|  |  |
| **Oligonucleotides used for sequencing and PCR for identifying the MFN2 KO mutation cells**  **(the size of PCR product was 683bp)** | |
| MFN2-For | CCATTGGGTCTGGCTAA |
| MFN2-Rev | GCAGTTCCCTTGTCTTCC |
|  |  |
| **Primers for cloning shRNAs (5’to 3’)** | |
| human MFN2 shRNA-#1-For | CcggGCAGGTTTACTGCGAGGAAATCTCGAGATTTCCTCGCAGTAAACCTGCTTTTTTg |
| human MFN2 shRNA-#1-Rev | aattcaaaaaaGCAGGTTTACTGCGAGGAAATCTCGAGATTTCCTCGCAGTAAACCTGC |
| human MFN2 shRNA-#2-For | CcggGTCAAAGGTTACCTATCCAAACTCGAGTTTGGATAGGTAACCTTTGACTTTTTTg |
| human MFN2 shRNA-#2-Rev | aattcaaaaaaGTCAAAGGTTACCTATCCAAACTCGAGTTTGGATAGGTAACCTTTGAC |
| human MFN2 shRNA-#3-For | CcggGCACTTTGTCACTGCCAAGAACTCGAGTTCTTGGCAGTGACAAAGTGCTTTTTTg |
| human MFN2 shRNA-#3-Rev | aattcaaaaaaGCACTTTGTCACTGCCAAGAACTCGAGTTCTTGGCAGTGACAAAGTGC |
| shRNA-Scramble-For | CcggTTCTCCGAACGTGTCACGTTTCAAGAGAACGTGACACGTTCGGAGAATTTTTTg |
| shRNA-Scramble-Rev | aattcaaaaaaTTCTCCGAACGTGTCACGTTCTCTTGAAACGTGACACGTTCGGAGAA |
|  |  |
| **MFN2 siRNA sequences (5’ to 3’)** | |
| MFN2 siRNA1# | CGGAGGAAGUGGAGAGGCATT |
| MFN2 siRNA2# | CCUCAAGGUUUAUAAGAAUTT |
| MFN2 siRNA3# | GCAAAGCUGCUCAGGAAUATT |
|  |  |
|  |  |

Supplementary Table 2. Detailed gene lists for the mitochondria PCR array and mitochondrial energy metabolism PCR Array.

| **Mitochondria genes (90)** | | | | **Mitochondrial Energy Metabolism genes (89)** | | | |
| --- | --- | --- | --- | --- | --- | --- | --- |
| AIFM2 | MFN1 | SLC25A15 | TIMM17A | ALDH2 | COX6B1 | NDUFA5 | NDUFS8 |
| AIP | MFN2 | SLC25A16 | TIMM17B | ARRDC3 | COX6C | NDUFA6 | NDUFV1 |
| BAK1 | MGME1 | SLC25A17 | TIMM22 | ASB1 | COX7A2 | NDUFA8 | NDUFV2 |
| BBC3 | MIPEP | SLC25A19 | TIMM23 | ATG5 | COX7A2L | NDUFAB1 | NDUFV3 |
| BCL2 | MPV17 | SLC25A2 | TIMM44 | ATP5A1 | COX7B | NDUFAF1 | PPA1 |
| BCL2L1 | MSTO1 | SLC25A20 | TIMM50 | ATP5B | COX8A | NDUFB10 | RNU11 |
| BID | MTX2 | SLC25A21 | TIMM8A | ATP5C1 | CYB561D1 | NDUFB2 | RPTOR |
| BNIP3 | NEFL | SLC25A22 | TIMM8B | ATP5F1 | CYC1 | NDUFB3 | SDHA |
| CDKN2A | NOS2 | SLC25A23 | TIMM9 | ATP5G1 | DNAH8 | NDUFB4 | SDHB |
| COX10 | OPA1 | SLC25A24 | TOMM20 | ATP5G2 | DNAJB1 | NDUFB5 | SDHC |
| COX18 | PARP1 | SLC25A25 | TOMM22 | ATP5G3 | EDN1 | NDUFB6 | SDHD |
| CPT1B | PMAIP1 | SLC25A27 | TOMM34 | ATP5H | FAS | NDUFB7 | SIRT1 |
| CPT2 | POLG | SLC25A3 | TOMM40 | ATP5I | GADD45B | NDUFB8 | TFAM |
| DNM1L | RHOT1 | SLC25A30 | TOMM40L | ATP5J | HSPA1A | NDUFB9 | TNF |
| FIS1 | RHOT2 | SLC25A31 | TOMM70A | ATP5J2 | HSPA1B | NDUFC1 | UQCR11 |
| FXC1 | RRM2B | SLC25A37 | TP53 | ATP5L | LRP5L | NDUFC2 | UQCRC1 |
| GRPEL1 | SFN | SLC25A4 | TSPO | ATP5O | MTOR | NDUFS1 | UQCRC2 |
| HSP90AA1 | SH3GLB1 | SLC25A5 | UCP1 | BCS1L | NDUFA1 | NDUFS2 | UQCRFS1 |
| HSPB1 | SLC25A1 | SOD1 | UCP2 | COX4I1 | NDUFA10 | NDUFS3 | UQCRH |
| HSPD1 | SLC25A10 | SOD2 | UCP3 | COX5A | NDUFA11 | NDUFS4 | UQCRQ |
| IMMP1L | SLC25A12 | STARD3 | UXT | COX5B | NDUFA2 | NDUFS5 |  |
| IMMP2L | SLC25A13 | TAZ |  | COX6A1 | NDUFA3 | NDUFS6 |  |
| LRPPRC | SLC25A14 | TIMM10 |  | COX6A2 | NDUFA4 | NDUFS7 |  |
